# Heterologous Expression of the Class IIa Bacteriocins, Plantaricin 423 and Mundticin ST4SA, in *Escherichia coli* Using Green Fluorescent Protein as a Fusion Partner

**DOI:** 10.3389/fmicb.2020.01634

**Published:** 2020-07-14

**Authors:** Ross Rayne Vermeulen, Anton Du Preez Van Staden, Leon Dicks

**Affiliations:** ^1^Department of Microbiology, Stellenbosch University, Stellenbosch, South Africa; ^2^Department of Physiological Sciences, Stellenbosch University, Stellenbosch, South Africa

**Keywords:** green fluorescent protein, plantaricin 423, mundticin ST4SA, heterologous expression, lactic acid bacteria, class IIa bacteriocins, fluorescent optimization, antilisterial activity

## Abstract

The antilisterial class IIa bacteriocins, plantaricin 423 and mundticin ST4SA, have previously been purified from the cell-free supernatants of *Lactobacillus plantarum* 423 and *Enterococcus mundtii* ST4SA, respectively. Here, we present the fusions of mature plantaricin 423 and mundticin ST4SA to His-tagged green fluorescent protein (GFP) for respective heterologous expression in *Escherichia coli*. Fusion of plantaricin 423 and mundticin ST4SA to His-tagged GFP produced the fusion proteins GFP-PlaX and GFP-MunX, respectively. Both fusion proteins were autofluorescent, circumvented inclusion body formation and lowered the toxicity of class IIa bacteriocins during heterologous expression. Not only did GFP-class IIa fusion stabilize heterologous expression and boost yields, the fluorescent intensity of GFP-PlaX and GFP-MunX could be monitored quantitatively and qualitatively throughout expression and purification. This robust fluorometric property allowed rapid optimization of conditions for expression and bacteriocin liberation from GFP via the incorporated WELQut protease cleavage sequence. Incubation temperature and IPTG concentration had a significant effect on bacteriocin yield, and was optimal at 18°C and 0.1–0.2 mM, respectively. GFP-MunX was approximately produced at a yield of 153.30 mg/L culture which resulted in 12.4 mg/L active mundticin ST4SA after liberation and HPLC purification. While GFP-PlaX was produced at a yield of 121.29 mg/L culture, evidence suggests heterologous expression resulted in conformation isomers of WELQut liberated plantaricin 423.

## Introduction

Peptides are ubiquitous in physiological systems where they fulfill functions which are fundamental to life. Within human physiology alone, peptides function as hormones, neurotransmitters, growth factors, ion channel ligands or antimicrobials which makes them an attractive therapeutic resource (Otvos and Wade, 2014; Recio et al., 2017; Pfalzgraff et al., 2018; Kumar, 2019). In terms of bioactivity, peptides generally show high selectivity, high efficacy and are normally well tolerated therapeutics due to their proteinaceous nature (Fosgerau and Hoffmann, 2015). However, peptides comprised of natural amino acids are not always ideal drug candidates due to their low oral bioavailability, poor stability and short plasma half-life (Otvos and Wade, 2014; Fosgerau and Hoffmann, 2015). Therefore, many challenges surround the use of antimicrobial peptides (AMP) as direct alternatives to classical antibiotic therapies. Fortunately, AMP producers are found throughout all kingdoms of life, which provides a wealth of chemical diversity and variable mechanisms of action over a wide range of environments (Brogden, 2005; Kumar et al., 2018). Using AMPs to combat resistance is more a task of elucidating peptides which provide a specific function within a specific environment and understanding the mechanisms which drive their activity. Therefore, any technique to improve the yield or spectrum of producible peptides is highly valuable, especially for uncharacterized AMPs identified through genome mining.

Bacteriocins are defined as AMPs that are ribosomally synthesized by bacteria and secreted into their environment to modulate the growth of other closely related bacterial species (Ennahar et al., 2000; Kotel’nikova and Gel’fand, 2002). Due to their proteinaceous nature, bacteriocins may not always be suitable antibiotic therapies, but have proven to be viable counterparts when microbial control is required in an environmental application. For example, fermented foods specifically produced by lactic acid bacteria (LAB), are examples of how bacteriocins can be used to help control unfavorable microbial contamination.

Species in the LAB phylum are Gram-positive, catalase negative, microaerophilic to anaerobic, asporogenous and low in GC content (Klein et al., 1998). Bacteriocin production is an important characteristic for LAB, which is evident in the wide range of bacteriocins produced from various classes and levels of complexity (Alvarez-Sieiro et al., 2016). The expression of multiple bacteriocin types or classes by a single LAB strain is common. This makes LAB metagenomes/genomes a fruitful mining ground for novel bacteriocins. Furthermore, LAB are native inhabitants of the gastrointestinal tract (GIT) and during GIT colonization bacteriocins produced by probiotic LAB undoubtedly play a role in shaping the local microbiome (Klein et al., 1998; Dicks and Botes, 2010; Brown, 2011; van Reenen and Dicks, 2011; Rupa and Mine, 2012).

**FIGURE 1:**
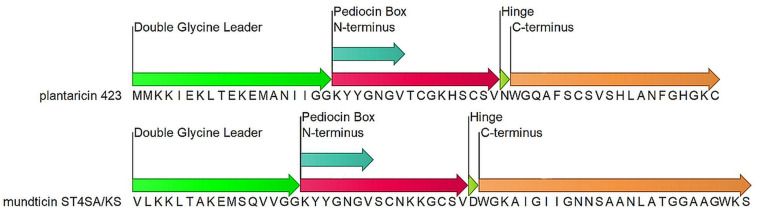
Precursor bacteriocin amino acid sequences for Plantaricin 423 and mundticin ST4SA. Indicated domains: double glycine leader (green), N-terminus (red), conserved YGNGV motif or pediocin Box (light Blue), Flexible hinge (light green) and the pore forming C-terminus (orange).

*Lactobacillus plantarum* 423 and *Enterococcus mundtii* ST4SA are LAB which produce the class IIa bacteriocins plantaricin 423 and mundticin ST4SA, respectively (Figure 1). *Lactobacillus plantarum* 423 was isolated from sorghum beer and harbors the plantaricin 423 precursor peptide gene, *plaA*, on the pPLA4 plasmid (Supplementary Table S1). Plantaricin 423 inhibits a number of food-borne pathogens such as *Bacillus cereus*, *Clostridium sporogenes*, *E. faecalis*, *Listeria* spp. and *Staphylococcus* spp. (van Reenen et al., 1998; Maré et al., 2006). *Enterococcus mundtii* ST4SA was isolated from soybeans and harbors the mundticin ST4SA pre-bacteriocin gene *munST4SA* within the munST4 operon (Supplementary Table S1). Mundticin ST4SA is active against *E. faecalis*, *Streptococcus pneumoniae*, *Staphylococcus aureus*, and *L. monocytogenes* (Granger et al., 2008).

Class IIa, or pediocin-like bacteriocins, are heat stable with post-translational modifications limited to disulfide bond formation (Ennahar et al., 2000; Cui et al., 2012). These peptides have high anti-listeria activity and a conserved N-terminal YGNGV motif, or pediocin box (Eijsink et al., 2002; Kjos et al., 2011; Cui et al., 2012; Lohans and Vederas, 2012; Alvarez-Sieiro et al., 2016). Peptides in this subclass are normally 25 to 48 amino acids in length which results in peptide masses less than 10 kDa. These peptides have a broad range of antimicrobial activity, predominantly targeting related LAB species and different strains of *Staphylococcus* and *Listeria*. Operons encoding class IIa bacteriocins are found natively in producer genomes, as introduced transposable elements or associated to small and large plasmids. While operon locations and organizations vary, they must always encode for proteins or elements which ensure bacteriocin regulation, immunity, maturation and extracellular translocation (Drider et al., 2006; Cui et al., 2012).

The precursor peptides of class IIa bacteriocins are made up of three domains; a leader peptide, N*-*terminal pediocin box and pore-forming C-terminus (Figure 1). Each domain is responsible for a different step in achieving antimicrobial activity via membrane poration. Most leader peptides in class IIa have a characteristic double glycine cleavage signal, however, some have a sec translocase secretion signal sequence. The double-glycine-type leader fused to the N-terminus of the core peptide produces an inert precursor peptide and functions as a secretory signal which is recognized by the operon-encoded ABC transporter. Cleavage of the leader peptide during bacteriocin transmembrane translocation from the producer cell is mediated by the ABC transporter and produces a mature, unstructured, extracellular bacteriocin (Ennahar et al., 2000; Drider et al., 2006; Nissen-Meyer et al., 2009; Cui et al., 2012; Bédard et al., 2018). Only upon interaction with a target membrane does the N-terminal pediocin box form a cationic three-stranded anti-parallel β-sheet-like structure, stabilized by a conserved disulfide bond (Fregeau Gallagher et al., 1997; Wang et al., 1999; Uteng et al., 2003; Haugen et al., 2005; Nissen-Meyer et al., 2009; Oppegård et al., 2015). The N-terminus of the core peptide mediates bacteriocin binding to target cell walls via docking to an extracellular loop of the IIC protein (MptC) from the sugar transporter mannose phosphotransferase system (Man-PTS) (Ramnath et al., 2000; Gravesen et al., 2002; Kjos et al., 2010). While the N-terminus associates with the MptC, a flexible hinge allows the C-terminal hairpin-like domain to transverse and penetrate the hydrophobic core of the target cell membrane (Figure 1; Ennahar et al., 2000; Eijsink et al., 2002; Drider et al., 2006; Nissen-Meyer et al., 2009; Kjos et al., 2011; Cui et al., 2012). Accumulation of the class IIa bacteriocin within a target cell wall results in pore formation and leakage of essential metabolites, protons and charged ions which dissipates the target cell’s transmembrane potential and pH gradient (Chikindas et al., 1993; Bennik et al., 1998; Montville and Chen, 1998). In addition, pediocin PA-1 was shown to cause the efflux of 2-α-aminoisobutyric acid a nonmetabolizable analog of alanine, and L-glutamate (Chikindas et al., 1993).

Like many other class IIa bacteriocins, plantaricin 423 and mundticin ST4SA have previously been purified from the native producer’s supernatant via an extensive purification process (van Reenen et al., 1998). For many bacteriocin classes like class IIa, peptides are usually chromatographically purified by taking advantage of their cationic and hydrophobic properties or smaller size (Lohans and Vederas, 2012). Not only is this process laborious with specific growth requirements, it can often result in poor or variable yields due to the inducible nature of bacteriocins, thus limiting their application (Carolissen-Mackay et al., 1997; Guyonnet et al., 2000; Lohans and Vederas, 2012). Furthermore, co-purification of active peptides is a possibility that can have a major downstream impact. Studies elucidating the mode of a bacteriocin’s regulation are inherently complex but may be doomed to fail when bacteriocins are chromatographically co-purified with contaminants such as autoinducing peptides or pheromones produced by the wild type strain.

Advances in chemically synthesizing peptides, including bacteriocins, has become a readily available option and solves these types of purification problems. Chemical synthesis has undoubtedly increased the application of bacteriocins in many areas but has limitations and may not work for every peptide. This is especially applicable for peptides which have only been observed *in silico* meaning their correct tertiary structures remain elusive (Henninot et al., 2018). Therefore, it may be prudent to continue the development of robust heterologous bacteriocin expression systems for research and high scale production applications.

Many class IIa bacteriocins have been produced using heterologous expression systems in *Escherichia coli*. The main objective of these expression systems has been to improve the large-scale production and rapid purification of class IIa bacteriocins. Purification of N-terminal His-tagged pediocin PA-1 expressed in *E. coli* resulted in low concentrations of biologically active bacteriocin (Moon et al., 2005). The majority of heterologously expressed His-tagged pediocin PA-1 was found in the insoluble fraction, most likely due to the bacteriocin’s solubility, size and the toxic effect on the *E. coli* host (Moon et al., 2005). The yields of heterologously expressed class IIa bacteriocins have been significantly improved when the mature peptide was fused to a larger, more soluble protein (Miller et al., 1998; Klocke et al., 2005; Moon et al., 2006; Beaulieu et al., 2007; Jasniewski et al., 2008; Liu et al., 2011). Although class IIa bacteriocins fused to thioredoxin are not secreted, this system produces the highest recorded yields ranging from 20 to 320 mg pure bacteriocin per liter of culture (Jasniewski et al., 2008; Liu et al., 2011). Thioredoxin is an 11.675 kDa highly soluble protein, which has a rigid solvent-accessible α-helix and can accumulate up to 40% of the total cellular protein in *E. coli* (LaVallie et al., 1993; Bell et al., 2013). Due to its size, solubility and cellular localization, thioredoxin aids in the expression and purification of class IIa bacteriocins by lowering toxicity and circumventing inclusion body packaging (LaVallie et al., 1993; Jasniewski et al., 2008; Liu et al., 2011; Bell et al., 2013; Kimple et al., 2013).

The Green Fluorescent Protein (GFP) gene, *mgfp5*, encodes a 26.908 kDa protein that forms a β-can cylindrical structure made up of 11 β-sheets surrounding a central axial-like α-helix producing fluorophore (Tsien, 1998; Zimmer, 2002). Posttranslational folding of GFP and chromophore formation is autocatalytic and does not require any cofactors except oxygen (Tsien, 1998; Zimmer, 2002). The folded protein is stable, soluble, non-toxic to *E. coli*, and has an excitation-emission autofluorescence at 488 and 509 nm, respectively. These characteristics make GFP applicable in molecular biology as a fluorescent cell marker, reporter gene or fusion tag (Tsien, 1998; Zimmer, 2002). The GFP gene *mgfp5* can be fused to class IIa bacteriocins in the same manner as the thioredoxin gene. Therefore, coupling sub-class IIa bacteriocins to GFP may provide many of the same benefits as thioredoxin but with the additional benefit of monitoring protein expression fluorometrically *in vivo* and in real time.

Presented here is the development of a fluorescent expression system to produce active plantaricin 423 and mundticin ST4SA using GFP as a fusion partner in *E. coli* BL21 (DE3). Furthermore, optimization approaches for expression, purification and cleavage using the fluorescent property of GFP is described.

## Results

### GFP-Bacteriocin Fusion Constructs

In order to take advantage of GFP as a fusion partner, the genes encoding mature plantaricin 423 and mundticin ST4SA were fused to the C-terminus of His-tagged GFP in the pRSF-GFP-PlaX and pRSF-GFP-MunX plasmid constructs, respectively (Supplementary Figures S5a,b). The newly generated GFP-fusion proteins, GFP-PlaX and GFP-MunX were successfully expressed in *E. coli* while retaining the autofluorescent properties of GFP. Furthermore, the inclusion of the WELQut protease cleavage site (WELQ), between GFP and the respective bacteriocins, allowed for liberation of active bacteriocin (Supplementary Figures S5a,b). After initial success, we further optimized expression using GFP-MunX.

### Optimization of GFP-MunX Expression in *E. coli* BL21

The fluorescent properties of GFP was used to evaluate the optimal expression conditions for increased yield production in terms of fluorescent output. This included different temperatures, expression times and IPTG concentrations, respectively.

#### Incubation Temperature Optimization for GFP-MunX Expression

**FIGURE 2:**
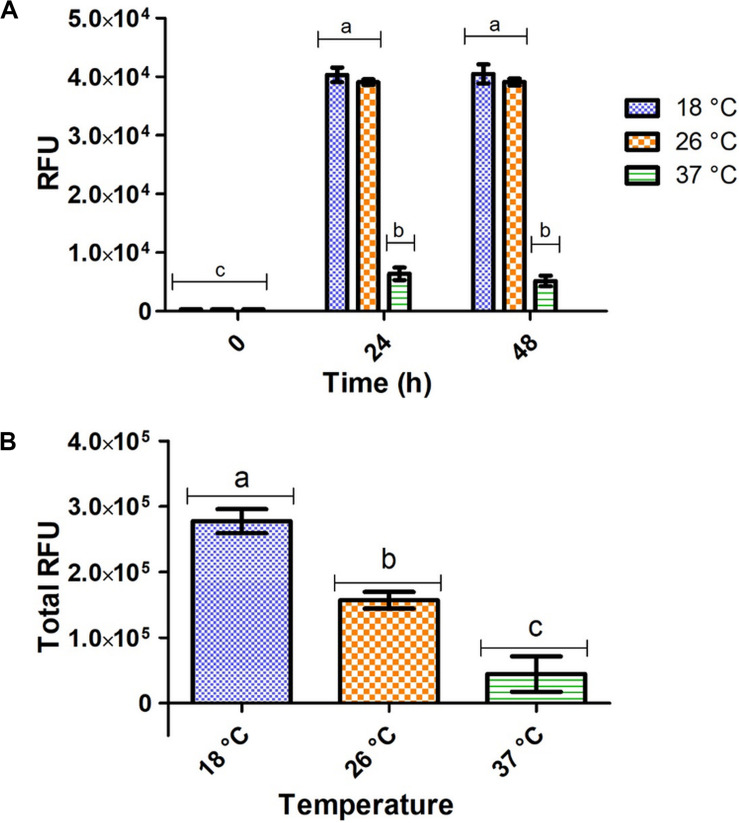
Fluorometric intensity of *E. coli* pRSF-GFP-MunEx expressing GFP-MunEx at 18, 26, and 37°C. **(A)** *In vivo* fluorometric measurements after 0, 24, and 48 h. **(B)** The total relative amounts of GFP-MunEx calculated after protein extraction and Ni-NTA purification of the 48 h expression. Fluorometric intensity measured in Relative fluorescent units (RFU). Dissimilar letters on bars indicates means which are significantly different from one another according to Bonferroni post-test (*P* < 0.05).

Significantly higher fluorescent intensity was measured *in vivo* at 18 and 26°C compared to 37°C after 24 and 48 h of expression, respectively (Figure 2A). However, these *in vivo* measurements do not consider total wet cell weight and do not accurately represent total target protein expression at each temperature. For measurement of total target protein expression in terms of relative fluorescence units (RFUs), the formula in Eq. 1 was used (Raw data found in Supplementary Table S2).

(1) $$\begin{array}{c} \text{Total RFU=}\frac{\text{RFUs of Ni-NTA purifiedeluent}}{\text{ wet cell weight used forpurification}}\\ \text{ × total cell weight} \end{array}$$

Total RFU production for GFP-MunX is represented in Figure 2B, where significantly higher RFUs were produced at 18°C. This fluorescent intensity was correlated to the presence of antimicrobial activity after cleavage using SDS-PAGE analysis (Supplementary Figure S2).

#### Fluorometric Optimization of IPTG Induction

**FIGURE 3:**
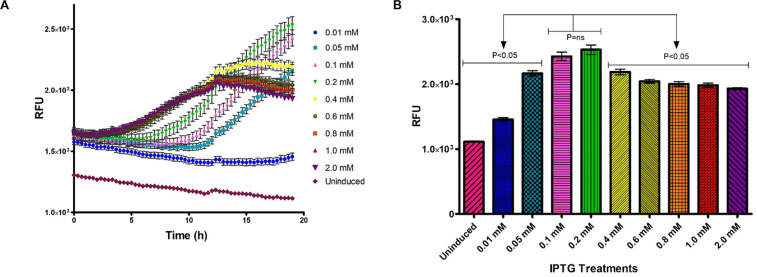
Optimization of IPTG concentration for GFP-MunX expression at 26°C over time, fluorescent intensity was measured in relative fluorescent units (RFUs). **(A)** *In vivo* RFU measurements captured every 25 min, were *n* = 3 (biological triplicates measured in technical triplicate), each point represents the mean with SEM indicated by error bars. **(B)** Mean RFU output comparison for GFP-MunX expression after 19 h incubation over the indicated range of IPTG concentrations. Arrows indicate Tukey’s multiple comparison test results identifying that 0.1 and 0.2 mM produce significantly higher RFU outputs from all other tests (*P* < 0.05).

The effect of IPTG concentration on heterologous protein expression was monitored *in vivo* and in real time using the Tecan Spark M10^TM^ (Tecan Group Ltd., Austria) kinetic incubation program and humidity cassette (Figure 3). Fluorometric output of induced samples increased with time and were significantly affected by the IPTG concentration used for induction (Figure 3A). IPTG concentrations of 0.1 and 0.2 mM induced significantly higher fluorescence after 18 h incubation at 26°C compared to other IPTG concentrations (Figure 3B).

### Upscaled Production of GFP-PlaX and GFP-MunX With Yield Approximation

In order to determine the effect of larger scale expressions on the yield of our GFP-fusion system we performed experiments under, respectively, optimized conditions using the Minifors 5L fermenter.

The *E. coli* pRSF-GFP-PlaX and pRSF-GFP-MunX fermentations were incubated at 18°C after 0.1 mM IPTG induction for 48 h in a 3 L fermentation volume. After extraction, Ni-NTA purification and buffer exchange of the GFP-PlaX and GFP-MunX proteins, 39 mL of GFP-PlaX and 42 mL of GFP-MunX eluent was obtained. After lyophilization of 1 mL GFP-PlaX and GFP-MunX, 12.96 mg and 17.96 mg residual mass was measured, respectively. From SDS-PAGE analysis the purity of GFP-PlaX and GFP-MunX are approximately 72 and 61%, producing approximate concentrations of 9.33 and 10.95 mg/mL, respectively (Supplementary Figure S3). At these purities, the approximate yield of GFP-PlaX and GFP-MunX was 121.29 mg/L of culture and 153.30 mg/L of culture, respectively.

### WELQut Cleavage Optimization

**FIGURE 4:**
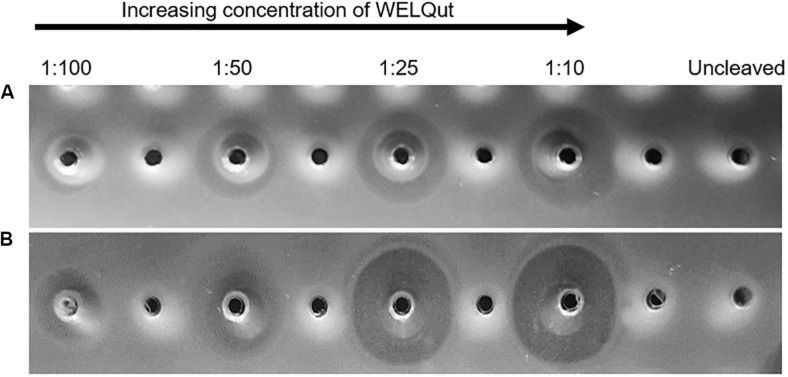
Antimicrobial activity of plantaricin 423 and mundticin ST4SA at various WELQut : sample ratios. Antimicrobial activity of plantaricin 423 **(A)** and mundticin ST4SA **(B)** cleaved from Ni-NTA purified GFP-PlaX and GFP-MunX proteins, respectively. Cleavage assessed using the spot plate technique against *L. monocytogenes*. Cleavage ratios of WELQut : sample (μL:μL) indicated on top of panel. Cleavage was performed at 28°C for 16 h. Post cleavage, 100 μL of GFP-PlaX **(A)** and 10 μL GFP-MunX **(B)** was spotted from each cleavage reaction. Uncleaved GFP-PlaX **(A)** and GFP-MunX **(B)** did not show antilisterial activity.

The WELQut cleavage reactions was optimized according to the stock concentrations of GFP-PlaX and GFP-MunX which were approximatly 9.33 mg/mL (12.96 mg/mL total protein) and 10.95 mg/mL (17.96 mg/mL total protein), respectively. The GFP-PlaX and GFP-MunX cleavage reactions were incubated at 28°C and sampled at time intervals of 2, 4, 8, and 16 h for a range of sample to WELQut ratios (Supplementary Figure S4). From these results, the cleavage ratios which produced maximal antilisterial activity for GFP-PlaX and GFP-MunX cleavage after 16 h was confirmed at a WELQut to sample ratio of 1:10 and 1:25 (μL:μL), respectively (Figure 4 and Supplementary Table S5b).

**FIGURE 5:**
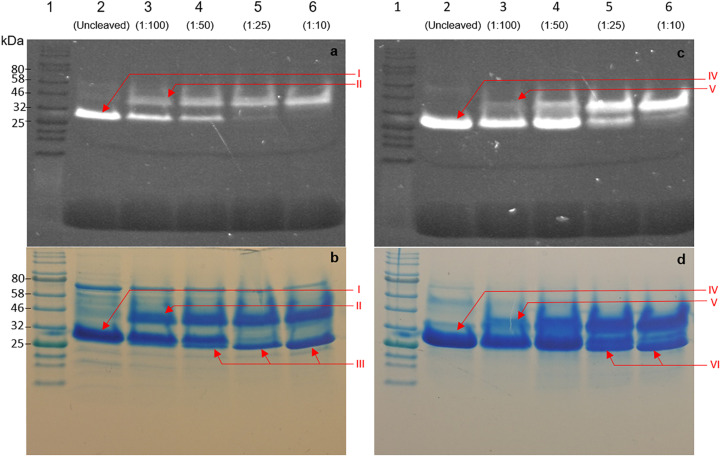
SDS-PAGE analysis of WELQut cleaved GFP-PlaX **(a,b)** and GFP-MunX **(c,d)**. **(a,c)** represent unstained SDS-PAGE gels fluorometrically photographed. **(b,d)** represent stained gels of **(a,c)**. Lane: 1 – Ladder, 2 – uncleaved sample, 3 to 6 WELQut cleavage samples, sample ratios indicated. Band I – Uncleaved GFP-PlantEx, II – putative WELQut and GFP complex, III – WELQut, IV – Uncleaved GFP-MunX, V – putative WELQut and GFP complex, VI – WELQut.

An important advantage of using GFP as a fusion partner is the ability to evaluate protease cleavage by visualizing migration patterns of fluorescent bands after electrophoretic separation. Determining optimal cleavage conditions in terms of activity of heterologously produced bacteriocin fusions is dependent on many variables. As such we confirmed the results for optimal cleavage by utilizing the maintained fluorescent properties of GFP after SDS-PAGE electrophoresis. Fluorescent bands were observed for GFP-PlaX, GFP-MunX, and GFP before and after cleavage, respectively (Figures 5a–c). These bands were then correlated to stained bands on the same SDS-PAGE gels (Figures 5b–d). An unexpected observation was the increase in size of GFP fluorescent bands of GFP-PlaX (band I) and GFP-MunX (band IV) after WELQut cleavage (band II and V, respectively). The fluorescent intensity of these larger bands (II and V) increases as increasing amounts of WELQut protease was added (columns 3 to 6 of Figure 5). This size increase of approximately 20 kDa might indicate the formation of a WELQut-GFP-bacteriocin complex forming upon WELQut liberation of plantaricin 423 and mundticin ST4SA, respectively.

**FIGURE 6:**
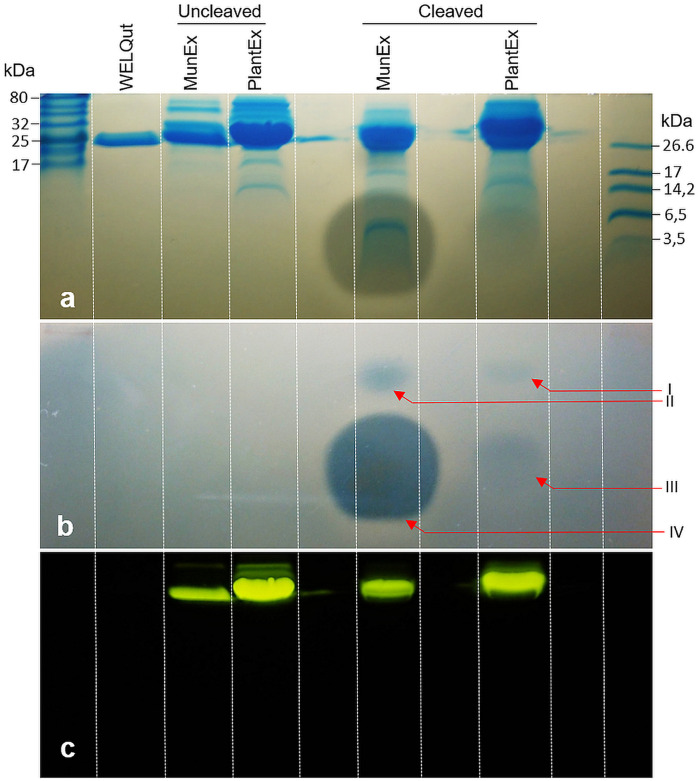
Observed post cleavage antilisterial activity of liberated mundticin ST4SA and plantaricin 423 separated by SDS-PAGE. **(a)** Superimposition of duplicate SDS-PAGE separations which indicates the size of bands showing antilisterial activity. **(b)** Antilisterial SDS-PAGE overlay showing activity post WELQut cleavage at locations correlating to I – GFP-PlaX, II – GFP-MunX, III – mundticin ST4SA and IV – plantaricin 423. **(c)** Location of fluorescent bands in **(a)**.

The intensity of the uncleaved GFP-PlaX and GFP-MunX fluorescent bands decreases as the WELQut : sample ratio increases (Figures 6a–c). Complete cleavage could be observed for GFP-PlaX (lane 6, Figure 5a) and corresponds to the highest spot activity observed (Figure 4A). Complete cleavage was not achieved for GFP-MunX, with a slight fluorescent band observed at the location of uncleaved GFP-MunX (lane 6, Figure 5c). Interestingly, the maximal spotted activity of GFP-MunX was equal for ratios 1:25 and 1:10 (Figure 4B) despite apparent difference in cleavage efficiency observed in SDS-PAGE (lanes 5–6, Figure 5c).

### Antimicrobial Activity Validation

While the respective bacteriocins were fused to GFP and produced a fluorescent complex, it was important to determine that antimicrobial activity was due to the liberated bacteriocin. The antimicrobial activity of plantaricin 423 and mundticin ST4SA post WELQut cleavage from GFP-PlaX and GFP-MunX proteins was investigated using SDS-PAGE under semi-native conditions (Figure 6). Using this method, the location of fluorescent GFP fusion proteins on the separated gel could be photographed and superimposed on the stained- and antilisterial gel overlay (Figures 6a–c). Antilisterial activity was observed as clear zones for the WELQut cleaved GFP-PlaX and GFP-MunX samples (Figures 6a,b). Antilisterial zones III and IV in Figure 6b correspond to the locations of mundticin ST4SA (4285 Da) and plantaricin 423 (3928 Da), respectively, indicating liberation of the core peptides from their respective GFP fusion partners. However, two additional zones of antilisterial activity (I and II in Figure 6b) were observed, which correspond to the approximate size and location of fluorescent GFP-MunX (31 874 Da) and GFP-PlaX (31 520 Da) (lane 5 and 6 of Figure 6c). From analysis of minimum inhibitory concentrations of cleaved GFP-MunX and –PlaX the BU/mL (bacteriocin units/mL) was determined to be 1600 and 83.33 BU/mL, respectively (Supplementary Figures S5, S6).

### Peptide Yields and LC-MS

Mundticin ST4SA and plantaricin 423 were cleaved from one milliliter of His-tag purified GFP-MunX and GFP-PlaX, respectively, under optimal cleavage conditions. Cleavage reactions were purified using high performance liquid chromatography (HPLC), single peaks were spot tested for antilisterial activity. Mundticin ST4SA activity was observed from a single peak while plantaricin 423 produced multiple active peaks with low levels of activity. The mundticin ST4SA fraction was lyophilized and the residual mass was weighed off. Optimal cleavage of GFP-MunX yielded 0.88 mg of active mundticin ST4SA, indicating that approximately 37.3 mg could be obtained from the 3 L fermentation corresponding to 12.4 mg/L mundticin ST4SA. The production yield of plantaricin 423 was not estimated due to multiple active peaks, showing weak activity, which is likely a result of different conformational isomers.

**FIGURE 7:**
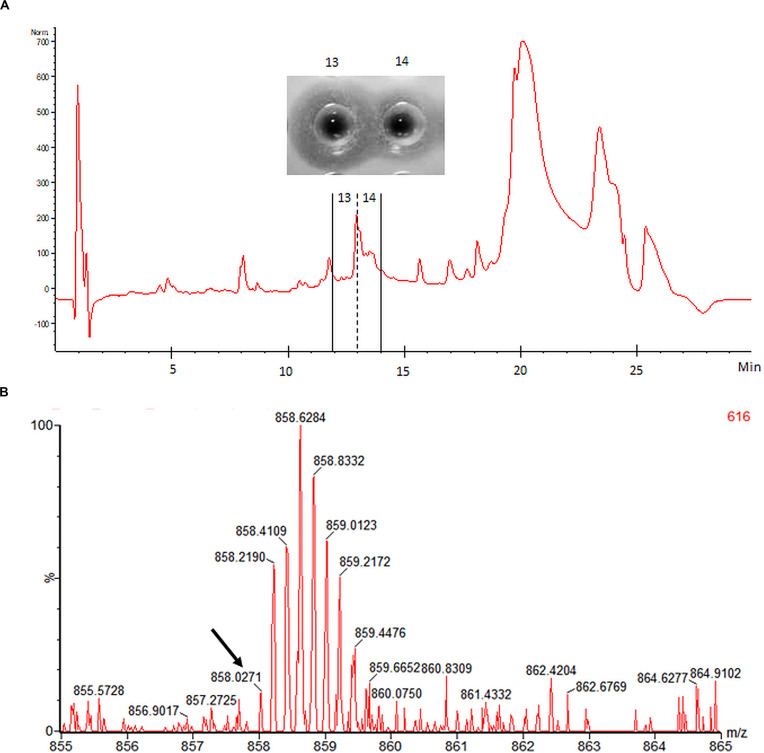
HPLC purification and accurate mass determination of mundticin ST4SA liberated from GFP-MunX. **(A)** HPLC fractionation of WELQut cleaved GFP-MunX mixture with antilisterial activity identified in fractions 13 and 14. **(B)** Segment of raw mass spectrum showing the observed m/z isotopic envelopes of mundticin ST4SA carrying +5 charges ([M+5H]^+5^ expected m/z 858.0232). The monoisotopic peak in **(B)** is indicated with an arrow and corresponds to an accurate mass measurement of 4258.1355 Da, which is in agreement with the exact mass of mundticin ST4SA with one disulfide bond (4285.0796 Da).

Electrospray ionization-MS performed on HPLC-purified mundticin ST4SA confirmed the presence of a peptide with a mass corresponding to mature mundticin ST4SA (Figure 7). While an accurate mass of 4285.1355 Da was determined for mundticin ST4SA from the isotopic envelope of the [M+5H]^+5^ species, the abundance of this charged species within the raw spectrum was low (Figure 7). However, the accurate mass measurement is in close agreement with the theoretical monoisotopic mass of 4285.0796 Da (equivalent to the formation of one disulfide bridge). Multiple attempts were made to determine the accurate mass of plantaricin 423, however, convincing data was not obtained.

## Discussion

Although expressing class IIa bacteriocins with a fusion partner redirects a portion of the metabolic flux away from bacteriocin synthesis, fusion increases overall yields by quenching the bacteriocin’s toxicity to *E. coli* (Liu et al., 2011). Thioredoxin has been a popular fusion partner for the heterologous expression of many subclass IIa bacteriocins. In an attempt to increase the usefulness of a bacteriocin fusion partner, we evaluated GFP as a fluorescent fusion partner for the heterologous expression of plantaricin 423 and mundticin ST4SA. Plantaricin 423 and mundticin ST4SA were purified from the soluble fraction due to the increased size and solubility when fused to GFP, as seen with thioredoxin fusion to other subclass IIa bacteriocins (Beaulieu et al., 2007; Jasniewski et al., 2008; Liu et al., 2011). The stabilizing effects provided by thioredoxin fusion in *E. coli* for class IIa bacteriocin expression is also provided by GFP, without disrupting its autofluorescent property.

Maintained fluorescence provided the advantage of clear visualization of the target proteins, GFP-PlaX and GFP-MunX, throughout the expression, extraction, purification, and analysis processes which allowed for rapid optimization and trouble shooting. Fluorescent intensity could function as a proxy to guide optimizations because it correlated to the amount of GFP fusion protein.

While previous studies have demonstrated that incubation temperature influences heterologous protein expression levels, to our knowledge, no study has considered this for the heterologous expression of class IIa bacteriocins (Sivashanmugam et al., 2009). Bacteriocins which have been expressed as fusion partners with thioredoxin include pediocin PA-1, carnobacteriocins BM1, and B2, divercin V41, enterocin P and piscicolin 124, with pediocin PA-1 also fused to mouse dihydrofolate reductase and enterocin A fused to cellulose-binding domain (Gibbs et al., 2004; Richard et al., 2004; Klocke et al., 2005; Cuozzo et al., 2006; Moon et al., 2006; Beaulieu et al., 2007; Jasniewski et al., 2008; Liu et al., 2011; Lohans and Vederas, 2012). These studies performed their expressions at 37°C or did not specify a temperature. While this study found that expression temperature significantly influenced the final GFP-bacteriocin yield in terms of RFU output. From this data it can be recommended that expression temperature is one of the first variables to be optimized for GFP-bacteriocin fusion expression. The ease of fluorometric optimization was also observed during IPTG treatments were real time *in vivo* results indicated that changes in IPTG concentrations significantly affected expression.

Evaluating what effect these variables will have on specific production rates would be a challenging and time-consuming task for non-fluorescent fusion partners. The GFP-bacteriocin fusion system boasts a great sense of confidence during heterologous expression of subclass IIa bacteriocins due to the high-quality data its convenient autofluorescent property provides. However, certain factors need to be taken into consideration. During expression temperature optimization, it was observed that the total wet cell weight must be taken into account when calculating total yield in terms of RFUs. *In vivo* measurements of GFP-MunX production indicate that there is no significant difference between the fermentations at 18 and 26°C. However, when the total RFUs produced were calculated after extraction and NI-NTA purification, nearly two times higher yields were obtained at 18°C as compared to 26°C. Without taking biomass into account, i*n vivo* fluorescent intensities can only be used to compare expression levels in real time as a means of coarse optimization. Therefore, *In vitro* (post purification) fluorescent intensities more accurately represent total target protein expression and are required for estimating yield.

Another major advantage of using GFP as a fusion partner is the ability to visualize fluorescence during SDS-PAGE analysis. Visualization indicated not only the location of the GFP fusion protein but also trends that occur during cleavage and liberation of the bacteriocin using WELQut protease. Therefore, loss of the GFP fusion protein at any stage of the extraction or purification process is immediately noticed. This robust fluorometric property of GFP can be attributed to the isolation of its fluorescent chromophore within the compact “β-can” cylindrical structure (Zimmer, 2002; Remington, 2011). The cylindrical structure provides resistance to sodium dodecyl sulfate (SDS), urea, beta-mercaptoethanol (BME) and dithiothreitol (DTT), however, its integrity is sensitive to pH and high temperature, hence the requirement for semi-native SDS-PAGE (Saeed and Ashraf, 2009; Krasowska et al., 2010).

The yields of most heterologously expressed subclass IIa bacteriocins, many of which are fused to thioredoxin, are rarely higher than tens of milligrams bacteriocin per liter of culture (Moon et al., 2006; Beaulieu et al., 2007; Liu et al., 2011; Lohans and Vederas, 2012). Under optimized conditions the GFP-PlaX and GFP-MunX fusion proteins were produced at approximately 121 mg/L of culture and 153 mg/L of culture, respectively. This would theoretically produce 15.08 and 20.56 mg/L mature plantaricin 423 and mundticin ST4SA assuming complete cleavage, respectively. After mature peptide cleavage, HPLC purification and lyophilization, 14.4 mg/L active mundticin ST4SA was recovered. Loss of sample during the purification process is expected and explains the difference between theoretical and observed mundticin ST4SA yields. Despite our promising yields, the highest yields for heterologous expression of class IIa bacteriocin were reported for carnobacteriocin B2 and BM1 fused to thioredoxin at 320 mg/L of culture (Jasniewski et al., 2008). Carnobacteriocin B2 and BM1 fusions were heterologously expressed in *E. coli* cultured in a fed-batch fermentation where pH, temperature and oxygen regulation were controlled. Jasniewski et al. (2008) reported that using a continuous supply of lactose for induction in the fed batch fermentation had a marked increase in heterologous expression. Future studies should consider expressing GFP as the fusion partner under such fed batch conditions at 18°C to assess whether GFP-subclass IIa fusion can improve on these yields. The yield of GFP-MunX and GFP-PlaX may also be improved by using alternative mechanisms of cell disruption such as alternative lysis buffers or mechanical methods.

A limiting factor observed in the current study was the liberation of active bacteriocin using WELQut protease. Cleavage at such an inefficient rate would be time consuming and costly, and therefore a major limiting factor for high scale production. An interesting observation was the apparent association of the WELQut protease with the respective fluorescent proteins post-cleavage causing an approximate 20 kDa size increase. Formation of a WELQut-GFP-bacteriocin complex is further supported by the presence of two unexpected zones of antilisterial activity observed post-cleavage at the location of fluorescent GFP-MunX and GFP-PlaX fusion protein bands. This WELQut-GFP-bacteriocin association might be indicative of a problematic cleavage as this phenomenon is unreported in the WELQut documentation or literature (Pustelny et al., 2014).

The WELQut protease is derived from the SpIB protease of *Staphylococcus aureu*s which is activated by proteolytic cleavage of an N-terminus signal peptide. Removal of the signal peptide allows subsequent formation of a characteristic hydrogen bond network (Pustelny et al., 2014). Pustelny et al. (2014) reports that signal peptide cleavage does not change the disposition of catalytic machinery nor does it perturb the hydrogen bond network in the vicinity of the catalytic site. The crucial elements within the active site are in place whether or not a signal peptide is attached, and does not depend on the signal peptide sequence (Pustelny et al., 2014). For this reason, Pustelny et al. (2014) postulates that interactions at the N-terminus affects the dynamics of SpIB as a whole which subsequently impacts substrate recognition and hydrolysis. Liberated plantaricin 423 and mundticin ST4SA may be interacting with SpIB at its N-terminus thus lowering its cleavage efficiency. Although, without fully understanding the mechanism of WELQut activation, hypothesizing how plantaricin 423 or mundticin ST4SA interfere with WELQut cleavage is difficult.

From the observations in the current study, and others, it is clear that the yields of heterologously expressed class IIa bacteriocins are not the biggest limiting factor for high scale production, but rather the liberation of the active core peptide. Fortunately, the maintained fluorescent property of GFP under semi-native SDS-PAGE conditions rapidly highlighted this bottleneck. The high yield of 320 mg/L obtained for carnobacteriocin B2 liberated from thioredoxin, was achieved using cyanogen bromide which unfortunately produces toxic samples that may limit downstream applications (Jasniewski et al., 2008). While studies like this one, which used a protease to liberate the subclass IIa bacteriocin from the fusion partner, are yet to achieve more than tens of milligrams pure bacteriocin per liter of culture (Moon et al., 2006; Beaulieu et al., 2007; Liu et al., 2011; Lohans and Vederas, 2012). This lower efficiency paired with the high cost of commercial proteases renders the approach unfeasible for high scale production. Using a bacteriocin’s native protease for fusion cleavage may lower costs and avoid any incompatibility issues as seen in this study with a potential increase in efficiency (Montalbán-López et al., 2018; Van Staden et al., 2019). In this regard the fluorescent property of GFP may prove a useful tool in identifying novel cleavage approaches or proteases effective at cleaving hydrophobic peptides containing disulfide bonds like subclass IIa bacteriocins.

Accurate mass determination for mundticin ST4SA and plantaricin 423 liberated from their GFP fusion partners was challenging. The determined accurate mass of 4285.1335 Da for mundticin ST4SA is in close agreement with the expected mass (4285.0796 Da) when one disulfide bond is formed. However, with the given concentration of mundticin ST4SA a relatively low abundance was observed during MS analysis.

Plantaricin 423 liberated from GFP-PlaX was not detected during MS analysis. Plantaricin 423 is similar to pediocin PA-1 as it has four cysteine residues which allows for the formation of two disulfide bonds. Different arrangements of the two bonds produce three conformational isomers of pediocin PA-1 with varying specific activities against *Listeria* (Oppegård et al., 2015; Bédard et al., 2018). This incorrect folding of pediocin PA-1 during heterologous expression is the result of an absent thioredoxin gene (Oppegård et al., 2015; Bédard et al., 2018). The thioredoxin gene, *papC*, is encoded by the native pediocin operon to guide correct disulfide bond formation for optimal specific activity and is also found in the *pla* operon (Mesa-Pereira et al., 2017). The low specific activity observed for liberated plantaricin 423 compared to mundticin ST4SA, in terms of RFUs, provides more evidence for the presence of conformational isomers.

Dividing the liberated mass of plantaricin 423 into different conformational isomers is exacerbating the difficulties experienced during LCMS analysis. These conformational isomers elute from the HPLC column in different fractions, which decreases the concentration of plantaricin 423 available for downstream analysis. Furthermore, activity was used to determine which fractions are submitted for MS, therefore fractions containing inactive plantaricin 423 were excluded altogether. This partitioning of the plantaricin 423 mass coupled with the potential for low abundance of charged species, may explain why plantaricin 423 was not detected during MS analysis. Modifications would need to be made to the current GFP-PlaX expression system to ensure correct disulfide bond formation before determining the production yield and accurate mass of plantaricin 423. In this regard, future studies should consider the co-expression of accessory proteins, such as the operon encoded thioredoxin proteins, to direct correct disulfide bond formation when conformational isomers are possible (Mesa-Pereira et al., 2017).

## Conclusion

Fusion to GFP stabilized expression, simplified purification by reducing peptide hydrophobicity and assigned a fluorometric tag to the target protein. Not only does this autofluorescent property make handling the target peptide more intuitive but it provides a high degree of confidence and reproducibility during heterologous peptide expression and purification. Furthermore, because fluorescence correlates to the amount of target protein and specific activity, optimization of most steps is rapid and convenient which ultimately accelerates the rate of progress. Our goal here was to show that fluorescence produced by GFP can be used to optimize heterologous expression. Future studies should evaluate the relationship between specific activity and the fluorescent coefficient, and its usefulness or implications, and on what scale this occurs. With the development of inline fluorescent monitoring technologies, future studies should also identify if time, temperature and IPTG concentration have compounding effects to further boost yield. However, the yields for plantaricin 423 and mundticin ST4SA produced with this GFP-fusion system will benefit greatly by improving both cleavage efficiency and disulfide bond formation. Finally, novel subclass IIa bacteriocins can be easily identified by their conserved YGNGV motif and produced with a high degree of process certainty using the GFP-fusion approach described here. However, subclass IIa bacteriocins which contain more than two cysteine residues may need additional accessory genes and therefore modifications to the system presented here.

Purifying hydrophobic peptides for bioactivity characterization is a challenging task especially under the non-native conditions experienced during heterologous expression. However, the antilisterial activity of subclass IIa bacteriocins is easily assayed, which conveniently demonstrated here that GFP is an effective and readily optimizable peptide-fusion partner. The extent to which GFP can function as a fusion partner for heterologous expression of cationic, hydrophobic peptides with toxic effects to *E.* coli, should be further explored. Recently, it has been demonstrated that class I and class II lanthipeptides can still undergo posttranslational modification while fused to the C-terminal of GFP (Ongey et al., 2018; Si et al., 2018; Van Staden et al., 2019). These studies further promote fusion to GFP as an elegant approach to improve the heterologous expression of modified or unmodified cationic, hydrophobic peptides in *E. coli* while maintaining their bioactivity. Therefore, if GFP-fusion can provide more confidence during heterologous expression and purification of various antimicrobial peptides, GFP may become the fusion partner of choice for peptides with much broader bioactivities.

## Materials and Methods

### Materials

Detailed information on materials and manufacture details are listed in the Supplementary Material.

### Bacterial Strains and Culture Conditions

All bacterial strains used in this study can be found in Supplementary Table S8. The LAB, *Lactobacillus plantarum* 423 and *Enterococcus mundtii* ST4SA were cultured on De Man, Rogosa and Sharpe (MRS) media, at 37°C without agitation. Luria Bertani (LB) medium, supplemented with 1.2% agar for solid medium, was used to culture *Escherichia coli* BL21 (DE3) during molecular cloning protocols. *Listeria monocytogenes* EGD-e was cultured on Brain Heart Infusion (BHI) media supplemented with 7.5 μg/mL Chloramphenicol. Terrific broth was used for the expression of heterologous proteins in recombinant *E. coli* BL21 (DE3) (Shi et al., 2011). For the selection and maintenance of pRSFDuet-1 (Novagen) derived plasmids, growth media were supplemented with 50 μg/mL kanamycin (Sigma).

### Molecular Techniques

DNA analysis, manipulation, and plasmid cloning were performed according to Sambrook et al. (1989). Genomic DNA and plasmid DNA isolations from *L. plantarum* 423 and *E. mundtii* ST4SA were performed according to Moore et al. (Ausubel et al., 2003) and O’Sullivan and Klaenhammer (1993), respectively. Plasmid DNA extractions from *E. coli* were performed using the PureYield^TM^ Plasmid Miniprep System according to the manufacturer’s instructions.

T4 DNA ligase and restriction enzymes (RE) were used according to the manufacturer’s instructions. Polymerase chain reaction (PCR) amplifications were performed using Q5 high-fidelity PCR DNA polymerase according to manufacturer’s instructions in a GeneAmp PCR system 9700 (ABI, Foster City, CA, United States).

Oligonucleotides were designed using the CLC main workbench program (CLC bio, Aarhus, Denmark). DNA sequencing was performed by the Central Analytical Facilities (CAF) at the University of Stellenbosch, South Africa.

Agarose gel electrophoresis was used for the analysis and purification of RE digested DNA fragments in TBE buffer at 10 V/cm using the Ephortec^TM^ 3000 V (Triad Scientific, Manasquan United States) power supply (Ausubel et al., 2003). Excised gel DNA fragments were purified using the Zymoclean^TM^ gel DNA recovery kit.

### GFP-Bacteriocin Fusion Plasmid Construction and Primer Design

The pRSFDuet-1 vector was used for the β–D–1-thiogalactopyranoside (IPTG) induction of the heterologously expressed His-tagged GFP-bacteriocin fusions in this study (Shi et al., 2011). The N-terminal of *mgfp5* (GFP) was fused to a hexahistidine tag in pRSFDuet-1 which was under the transcriptional control of the T7 promoter (Supplementary Figure S7). The double-glycine leader sequences from mundticin ST4SA and plantaricin 423 were excluded so that the core peptide’s N-terminus was fused to the C-terminus of GFP. The WELQut protease recognition amino acid cleavage sequence was introduced between GFP and the respective core peptide sequences (Supplementary Figure S5). This site allowed for posttranslational cleavage and liberation of the core peptides using the WELQut protease.

The GFP_Bam_Fwd forward primer installed a *BamH*I site 5′ of *mgfp5* (GFP gene) for cloning into pRSFDuet-1 (Supplementary Table S6). The GFP_WELQ_Rev reverse primer extended the *mgfp5* sequence with an *Age*I restriction site followed by the DNA sequence encoding the WELQ amino acid sequence (recognition sequence for WELQut protease), followed by a *PstI* site (Supplementary Figure S5). The PlaX_Pst_Fwd/PlaX _Hind_Rev and MunX_Pst_Fwd/MunX_Hind_Rev primer sets annealed to the mature plantaricin 423 (*plaA*) and mundticin ST4SA (*munST4SA*) gene sequences, respectively, (Supplementary Table S3). These primer sets added 5′ *PstI* and 3′ *Hind*III restriction sites for cloning mature plantaricin 423 (*plaA*) or mundticin ST4SA (*munST4SA*) genes as GFP fusions in pRSFDuet-1, represented as the “core peptide” in Supplementary Figure S5. Detailed cloning procedure for the construction of pRSF-GFP-PlaX and pRSF-GFP-MunX may be found in the Supplementary Material.

### Overexpression of GFP-Bacteriocin Fusion Proteins in *E. coli* BL21 (DE3)

Starter cultures of 30 mL LB broth containing 50 μg/mL kanamycin were inoculated with respective *E. coli* BL21 (DE3) transformants containing pRSF-GFP-PlaX or pRSF-GFP-MunX constructs. The starter cultures were incubated at 37°C for 12 h with constant agitation. Starter cultures were used as an inoculum for the expression of GFP-PlaX and GFP-MunX, respectively, (1% v/v). At an OD_600_ of 0.6–0.65, expression of the respective GFP fusion proteins was induced using 0.1 mM IPTG. Upscaled production in a 5 L fermenter (Minifors, Infors AG, CH – 4103 Bottmingen/Basel, Switzerland) is detailed in the Supplementary Material.

### Ni-NTA Purification of GFP-MunX and GFP-PlaX Proteins

Induced cells were harvested by centrifugation at 8 000 *g* for 20 min at 4°C. The supernatant was discarded, and the cell pellet was resuspended in 15 mL/g wet weight SB buffer supplemented with 1 mg/mL lysozyme and incubated with agitation at 8°C for 45 min (Buffer compositions can be found in Supplementary Table S7). After incubation, the lysed cells were subjected to sonication (50% amplitude, 2 s pulse, 2 s pause, 6 min) using the Omni Ruptor 400 (Ultrasonic Homogenizer, Omni International). RNaseI and DNaseI were added to a final concentration of 10 and 5 μg/mL, respectively, and the lysate incubated at room temperature for 15 min. The cell lysate was then centrifuged for 90 min at 20 000 *g* at 4°C; the cell-free supernatant was collected. Imidazole was added to the cell-free supernatant to a final concentration of 10 mM.

The His-tagged GFP-bacteriocin fusion proteins, GFP-PlaX and GFP-MunX, were purified with immobilized metal affinity chromatography (IMAC) using the Ni-NTA superflow resin, according to the Qiagen expressionist handbook’s instructions for batch purification. His-tagged proteins were purified using 25 mL of Qiagen Ni-NTA superflow resin in combination with a 26 mm diameter adjustable length flash column (Glasschem, Stellenbosch, South Africa). The Ni-NTA superflow resin was equilibrated in SB10 (SB buffer containing 10 mM imidazole) buffer and then added directly to the cell-free supernatant. The slurry was gently agitated for 2 h at 8°C using a shaker, after which the Ni-NTA superflow resin slurry was packed into the column. The ÄKTA purifier system (Amersham, Biosciences) was used for IMAC purification according to the following program; 5 column volumes (CV) SB10 (2% B buffer where A is SB and B is SB500), washed with 10 CV of SB20 (4% B buffer), elution occurred in approximately 40 mL of SB500 (100% B buffer). Eluted proteins were detected at 254 and 280 nm, respectively. Eluted His-tagged proteins were desalted using size exclusion chromatography. The ÄKTA purifier system was used in conjunction with Sephadex G25 resin packed into column (16 × 65 mm, GE Healthcare) for exchanging the sample from SB500 to WELQut cut buffer.

### Incubation Temperature Optimization for GFP-MunX Expression

Only the GFP-MunX fermentation was temperature optimized as cleaved GFP-MunX had a higher specific activity. The GFP-MunX expression temperature optimization was performed at 18, 26, and 37°C with three biological repeats of *E. coli* BL21 (DE3) harboring the pRSF-GFP-MunX plasmid. The three biological repeats of *E. coli* pRSF-GFP-MunX were used to inoculate three 400 mL flasks of terrific broth containing 50 μg/mL kanamycin. The cultures were grown at 37°C until induction using 0.1 mM IPTG at an OD_600 nm_ of 0.6–0.65. Each 400 mL culture was split into three 100 mL cultures that were incubated at 18, 26, and 37°C for 48 h, respectively.

To measure *in vivo* GFP-MunX expression, 1 mL samples from each flask were collected in triplicate at the time of induction; samples were collected again at 24 and 48 h and frozen at −80°C. After collection, samples were thawed, centrifuged and washed twice with potassium phosphate buffer (pH 7.4). The *in vivo* expression of GFP-MunX was fluorometrically measured in relative fluorescent units (RFUs) at 488 nm (excitation) and 509 nm (emission) using a Tecan Spark M10^TM^ (Tecan Group Ltd., Austria).

After 48 h, the total GFP-MunX RFU production for each sample was calculated by harvesting the induced cells from 80 mL of culture (centrifugation 8000 × *g* for 20 min at 4°C). The mass of each cell pellet was then measured before resuspension in 15 mL/g wet weight SB buffer. The GFP-MunX in each sample was extracted and purified using IMAC as described previously. Briefly, 5 mL from each cell-free lysate was purified using 5 ml Ni–NTA Superflow cartridges. The RFUs of each Ni-NTA purified GFP-MunX sample was measured using the Tecan M10^TM^ Spark (Tecan Group Ltd., Austria). The RFUs/g were then calculated for each sample by dividing the measured RFUs by the equivalent wet weight (g) of cells lysed for purification (i.e., grams of lysed cells in 5 mL). The total RFUs produced for each sample was calculated by multiplying the RFUs/g by the total wet weight of cells harvested in each sample.

### Optimization of IPTG Concentration for Induction

Fluorometric intensity was used to optimize IPTG induction concentration for GFP-MunX expression using the Tecan Spark M10^TM^’s (Tecan Group Ltd., Austria) kinetic incubation program and humidity cassette in a 96 well microtiter plate. Three biological repeats of *E. coli* BL21 (DE3) harboring the pRSF-GFP-MunX plasmid were inoculated into three 1 L Erlenmeyer flasks containing 200 mL filter sterilized terrific broth supplemented with 50 μg/mL kanamycin, and incubated at 37°C. Once an OD_600 nm_ of 0.55 was reached, each culture was chilled in an ice bath. Culture aliquots were then incubated with 0.01, 0.05, 0.1, 0.2, 0.4, 0.6, 0.8, 1, and 2 mM IPTG (final concentration) in triplicate in a 96 well microtiter plate. The microtiter plate was incubated within a humidity chamber by the Tecan Spark M10^TM^ at 26°C for 20 h. Every 20 min the microtiter plate was shaken for 30 s, allowed to settle for 10 s, RFUs were then measured at 509 nm (emission) after excitation at 488 nm.

### Concentration Estimation

One milliliter of Ni-NTA purified and buffer exchanged GFP-PlaX and GFP-MunX eluents were lyophilized and analytically weighed off in triplicate to estimate total protein mass. The purified GFP-PlaX and GFP-MunX samples were electrophoretically separated using tricine SDS-PAGE to estimate sample purity (Schägger and von Jagow, 1987). To avoid saturation during Coomassie staining the 10× dilutions of GFP-PlaX and GFP-MunX were used to estimate protein purity (Supplementary Figure S3). Gel analyzer 2010a^1^ was used to determine the pixel density of each stained band in respective lanes and used to estimate sample purity (Lazer and GelAnalyzer, 2010). WELQut cleavage optimization.

Cleavage parameters were optimized using a modification of the method supplied by Thermo Fisher Scientific for the WELQut protease. The WELQut-to-sample ratios were set to 1:100, 1:50, 1:25, 1:5 (v/v) for 50 μL samples of GFP-PlaX and GFP-MunX, respectively, and diluted to a final volume of 250 μL in WELQut cut buffer (Supplementary Table S7). The corresponding units of WELQ to approximately 466.5 μg of GFP-PlaX was 2.5, 5, 10, and 50 U, respectively. The corresponding units of WELQ to approximately 547.5 μg of GFP-MunX was 2.5, 5, 10, and 50 U, respectively.

### Antimicrobial Activity Assays

Antimicrobial activity of plantaricin 423 and mundticin ST4SA was assessed against *Listeria monocytogenes* EDG-e grown on Brain Heart Infusion media (BHI) containing 7.5 μg/mL chloramphenicol. The spot plate method was performed on solid medium (1% w/v agar) seeded with *Listeria monocytogenes* EDG-e. SDS-PAGE separations were assayed for activity by casting the polyacrylamide gel in an agar bilayer seeded with *Listeria monocytogenes* EDG-e. Before casting, the poly acrylamide gels were fixed for 20 min in a 25% isopropanol, 10% acetic acid fixing solution and then rinsed 3 × 15 min with sterile ultra-pure water.

The MIC of GFP-MunX and -PlaX against *L. monocytogenes* EDG-e was determined using a 96 well microtiter plate assay at various concentration of cleaved GFP-MunX and GFP-PlaX in technical and biological triplicate. An overnight culture of *L monocytogenes* EDG-e was used to inoculate BHI broth containing 7.5 μg/mL chloramphenicol at 1% v/v. GFP-MunX and GFP-PlaX was cleaved at the predetermined optimal WELQut to sample ratios of 1:25 and 1:10, respectively. Serial dilutions of cleaved GFP-MunX and –PlaX stock concentrations were made (Supplementary Figures S5, S6). Due to the differences in GFP-MunX and -PlaX concentration and differences in mundticin ST4SA and plantaricin 423 specific activity, 10 μL of each GFP-MunX samples and 20 μLGFP-PlaX samples was added to 190 and 180 μL BHI broth containing *L. monocytogenes* EDG-e in a 96 well microtiter plate, respectively. Absorbance readings (595 nm) were recorded at times 0 and 18 h. Listerial inhibition was expressed as I = 1 – (A_m_/A_0_), where A_m_ is the sample absorbance and A_0_ the control (blank) absorbance at 595 nm (Cabo et al., 1999). One bacteriocin unit (BU) is defined here as the minimum amount of bacteriocin required to inhibit at least 50% of the indictor strain’s growth in 200 μL culture volume (Cabo et al., 1999).

### HPLC and LC-ESI-MS

For yield calculation of MunX, GFP-MunX was digested with WELQut protease as described elsewhere and injected onto a Discovery BIO Wide Pore C18 HPLC column (10 μm, 250 × 10 mm, Sigma-Aldrich). The sample was separated using a gradient of 10–90% Solvent B (Solvent B: 90% Acetonitrile/10% MilliQ, 0.1% TFA) over 12 min. Active peaks were collected and lyophilized in order to determine residual mass.

For ESI-MS analysis cleaved GFP-MunX was heated at 90°C for 10 min and cetrifuged (12000 × *g*, 10 min). The supernatant was subsequently injected onto a Hypersil Gold C18 HPLC column (5 μm, 4.6 × 100, Thermo Fisher Scientific). The sample was separated using a gradient of 10–50% Solvent B (Solvent B: Acetonitrile, 0.1% TFA) over 10 min. Peaks were collected and tested for activity as described elsewhere. Active peaks were concentrated using a SpeedyVac vacuum concentrator before ESI-MS analysis (CAF, Stellenbosch, South Africa).

### Statistical Analysis

Statistical analysis was performed using Graph Prism Version 3.0 (Graph Pad Software, San Diego, CA). Data generated from the effect of incubation temperature, time and IPTG induction concentration on heterologous expression of GFP fusion constructs was represented as the mean with stand error of mean (SEM). Bonferroni post-tests was performed after a two-way ANOVA on *in vivo* incubation temperature optimization data to identify significant treatments over time. Tukey’s Multiple Comparison Test was performed after a one-way ANOVA on the final time point data from IPTG optimization data only, to identify significant treatments. *P*-values < 0.05 were considered significant.

## Data Availability Statement

The datasets generated for this study are available on request to the corresponding author.

## Author Contributions

RV and AV conceptualized the study, performed the experiments, analyzed the data, and wrote the manuscript. LD was responsible for funding acquisition, aided in conceptualization of the study, and writing of the manuscript. AV and LD supervised RV. All authors contributed to the article and approved the submitted version.

## Conflict of Interest

The authors declare that the research was conducted in the absence of any commercial or financial relationships that could be construed as a potential conflict of interest.

## Footnotes

1 http://www.gelanalyzer.com/
