# Supplementary material for: Heterologous Expression of the Class IIa Bacteriocins, Plantaricin 423 and Mundticin ST4SA, in Escherichia coli Using Green Fluorescent Protein as a Fusion Partner
Source: Front Microbiol. 2020 Jul 14;11:1634. doi: 10.3389/fmicb.2020.01634 (PMC7381239; doi:10.3389/fmicb.2020.01634)
Supplement: Supplementary file 1 [file Data_Sheet_1.pdf]

Supplementary Information

Heterologous expression of subclass IIa bacteriocins in Escherichia coli using green fluorescent protein as a fusion partner

Ross Rayne Vermeulen[1] , Anton Du Preez van Staden[1, 2] , and Leon Dicks[1]

[1] Department of Microbiology, Stellenbosch University, Matieland, South Africa
[2] Department of Physiological Sciences, Stellenbosch University, Matieland, South Africa

*Corresponding author:

Anton Du Preez van Staden

advstaden@outlook.com

Leon Dicks

LMTD@sun.ac.za

1. Materials

Bacterial growth media was from Biolab (Biolab, Merck, South Africa) while all buffer components, salts and glycerol were from Merck Millipore. Plasmid DNA extractions from *E. coli* were performed using the PureYield™ Plasmid Miniprep System (Promega) according to the manufacturer's instructions. T4 DNA ligase and restriction enzymes (RE) were purchased from New England Biolabs (New England Biolabs (NEB)) and used according to the manufacturer's instructions. Polymerase chain reaction (PCR) amplifications were performed using Q5 high-fidelity PCR DNA polymerase (NEB) according to manufacturer's instructions in a GeneAmp PCR system 9700 (ABI). Agarose gel DNA rrecovery was performed using the Zymoclean Gel DNA Recovery kit (Zymo Research Corporation). Oligonucleotides were designed using the CLC main workbench program (CLC bio) and purchased from Inqaba Biotechnical Industries. DNA sequencing was performed by the Central Analytical Facilities (CAF) at the University of Stellenbosch, South Africa. Imidazole, Trifluoroacetic acid (TFA), acetonitrile (LC-MS grade), Nickle Chloride (IMAC column regeneration), acrylamide/Bis-acrylamide 30% solution, Coomassie G250 and urea was from Sigma Aldrich. WELQut protease, lysozyme, BCA protein assay and PageRuler low range protein ladder was from ThermoFisher Scientific. Ni-NTA his tag columns and resin was from Qiagen and size exclusion resin was from GE Healthcare.

2. Construction of the plantaricin 423 and mundticin ST4SA GFP-bacteriocin expression vectors

The pTRKH3p15A-ErmGFP plasmid was used as a template for the PCR amplification of *mgfp5* using the GFP_Bam_Fwd/ GFP_WELQ_Rev primer set (Supplementary Table S6). The *mgfp5* amplicon was cloned using the cloneJET PCR cloning system according to the manufacturer's instructions (Thermo Scientific). Plasmid DNA was extracted from ampicillin resistant transformants and sequenced using the pJET1.2_Fwd and pJET1.2_Rev primer set (Supplementary Table S6).

Genomic DNA from *L. plantarum* 423 and *E. mundtii* ST4SA was used as templates to amplify mature plantaricin 423 and mundticin ST4SA bacteriocin genes using the GFP-PlaX_Pst_Fwd/GFP-PlaX_Hind_Rev and GFP-MunX_Pst_Fwd/GFP-MunX_Hind_Rev primer sets, respectively (Supplementary Table S6). The amplified bacteriocin genes were purified using the GeneJet PCR purification kit (Thermo Scientific) and digested with *Pst*I and *Hind*III. The digestion mixtures were purified again using the GeneJet PCR purification kit according to the manufacturer's instructions and used in subsequent cloning experiments.

The pJET-GFP plasmid was digested with *Bam*HI/*Pst*I; the pRSFDuet-1 vector was digested with *Bam*HI/*Hind*III. The linear pRSFDuet-1 vector and digested GFP fragment were purified using agarose gel electrophoresis, gel-excised and recovered. In one single ligation reaction, the *BamH*I/*Pst*I GFP fragment and *Pst*I/*Hind*III bacteriocin fragment was ligated into the linear pRSFDuet-1 vector (*Bam*HI/*Hind*III). The fragments were ligated using a Vector:Insert_GFP:Insert_Bacteriocin molar end ratio of 1:3:3. The resulting constructs, pRSF-GFP-PlaX, and pRSF-GFP-MunX, were used to transform chemically competent *E. coli* BL21 (DE3) cells. The pRSF-GFP-PlaX and pRSF-GFP-MunX plasmids were extracted from phenotypically green fluorescent, kanamycin (50µg/mL) resistant colonies of *E. coli* BL21 (DE3). The mature plantaricin 423 and mundticin ST4SA genes were sequenced in pRSF-GFP-PlaX and pRSF-GFP-MunX plasmids using the MCS1_Rev primer and confirmed to be correct (Supplementary Table S6).

3. Upscaled production of GFP-PlaX and GFP-MunX

Upscaled heterologous expression of the plantaricin 423 and mundticin ST4SA GFP fusion proteins was performed using a 5 L fermenter (Minifors, Infors AG; 3 L max recommended capacity). Terrific broth (2.7L), containing 0.005% antifoam 204 (Sigma-Aldrich), was prepared and autoclaved. Once cool, 300 mL of sterile 10x TB buffer and kanamycin (50 µg/mL final concentration) was aseptically added (Supplementary Table S7). The broth was heated to 37 °C, aerated at 1 L/min with sterile compressed air and stirred at 300 RPM. The pH and dissolved oxygen levels were not controlled.

The starter cultures of *E. coli* BL21 (DE3) pRSF-GFP-PlaX and pRSF-GFP-MunX were used as an inoculum at 1% v/v, for respective expressions. At an OD_{600nm} of 0.6 – 0.65, expression of the respective GFP fusion proteins was induced using 0.1 mM IPTG (Thermo-Fisher Scientific). Respective fermentations were then cooled to 18 °C and incubated for 48 h.

4. WELQut cleavage optimization

Cleavage parameters were optimized using a modified method from that supplied by the manufacturer. The WELQut-to-Sample ratios were set to 1:100, 1:50, 1:25, 1:5 (v/v) for 50 µL samples of GFP-PlaX and GFP-MunX, respectively, and diluted to a final volume of 250 µL in WELQut cut buffer (Supplementary Table S7). The approximate corresponding units of WELQ to 466.5 µg of GFP-PlaX was 2.5 U, 5 U, 10 U and 50 U respectively. The approximate corresponding units of WELQ to 547.5 µg of GFP-MunX was 2.5 U, 5 U, 10 U and 50 U respectively. Cleavage reactions were incubated at 28 °C, and 50 µL samples were collected at 2 h, 4 h, 8 h, and 16 h, respectively. Cleavage was assessed by the spot plate method using BHI solid medium (1% w/v agar) seeded with *Listeria monocytogenes* EGD-e.

5. Correlating antimicrobial activity to fluorescence

The fluorescent intensity of GFP-MunX and -PlaX was correlated to their respective antilisterial activities by measuring the fluorescent intensity of the respective serial dilutions used in the antilisterial MIC assay (Fig S5 and 6). Fluorescent intensities were measured on the Tecan Spark M10™ at 509 nm (emission) after excitation at 488 nm.

Supplementary Table S1 Biochemical properties of plantaricin 423 and mundticin ST4SA

**Bacteriocin (Producer)**	**Nucleotide Accession no.**	**Protein Accession no.**	**Precursor Peptide size (aa)**	**MP* size (aa)**	**MP Mass# (Da)**	**Accurate Mass**	**Operon accession no.**	**Ref**
Plantaricin 423 (*L. plantarum* 423)	AF304384.2	AAL09346.1	56	37	3928.74	N/A	AF304384.2	1
Mundticin ST4SA (*E. mundtii* ST4SA)	MN296285	QHN63927.1	59	43	4285.10	4285.135	N296285	2

*MP = Mature peptide
Theoretical mass (including the formation of disulfide bridges)
1 (van Reenen et al., 1998); 2 (Granger et al., 2008)

Supplementary Table S2 Total RFU produced from Ni-NTA purified GFP-MunX temperature optimization fermentations after 48h expression

Sample	Temperature (°C)	WCM* (g)	Resuspension volume	Ni-NTA Eluent fluorescence ϕ (RFU#)	RFU/g	Total RFU produced	Average total RFU per temperature	Standard error (Total RFU)	Standard deviation (Total RFU)
G18	18	2.47	37.05	33728	101286.2	250177.1	277 693.5	18 344.2	31 773.1
O18		2.39	35.85	37680	113153.1	270436			
Y18		2.5	37.05	41620	124986.9	312467.5			
G26	26	1.22	18.3	47975	144070.0	175765.5	156 734.1	12 738.8	22 064.2
O26		1.15	17.25	38381	115260.2	132549.3			
Y26		1.41	21.15	38233	114813.8	161887.5			
G37	37	1.38	11.4	23436	70378.3	97122.2	44 340.6	27 030.0	46 817.4
O37		0.8	20.7	11684	35089.1	28071.3			
Y37		0.76	12	3430	10300.3	7828.2			

* WCM = Wet cell mass
ϕ A wet cell weight equivalent to 0.333 g was used for each purification
RFU = Relative florescent units

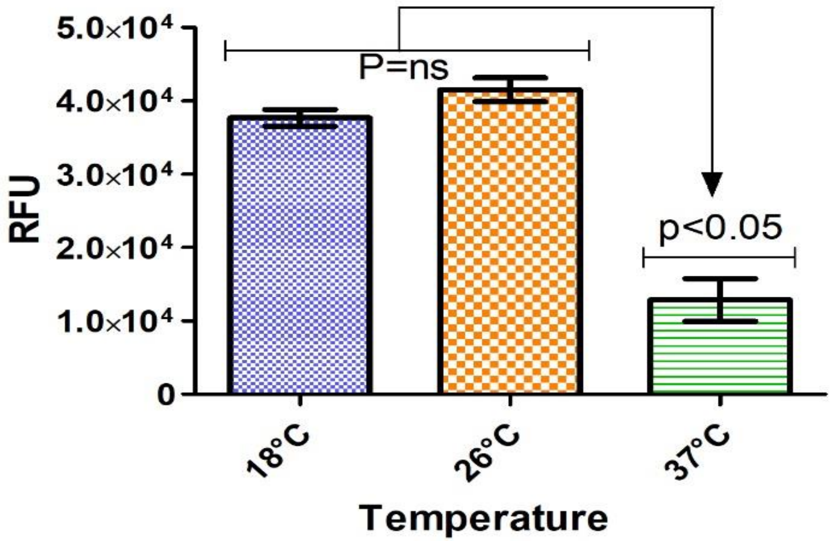

Supplementary Figure S1. Fluorometric intensity of GFP-MunEx after protein extraction and Ni-NTA purification from *E. coli* pRSF-GFP-MunX expressing GFP-MunX at 18 °C, 26 °C, and 37 °C for 48 h. Fluorescence was normalized according to cell resuspension volumes (Table S2 - Ni-NTA Eluent fluorescence).

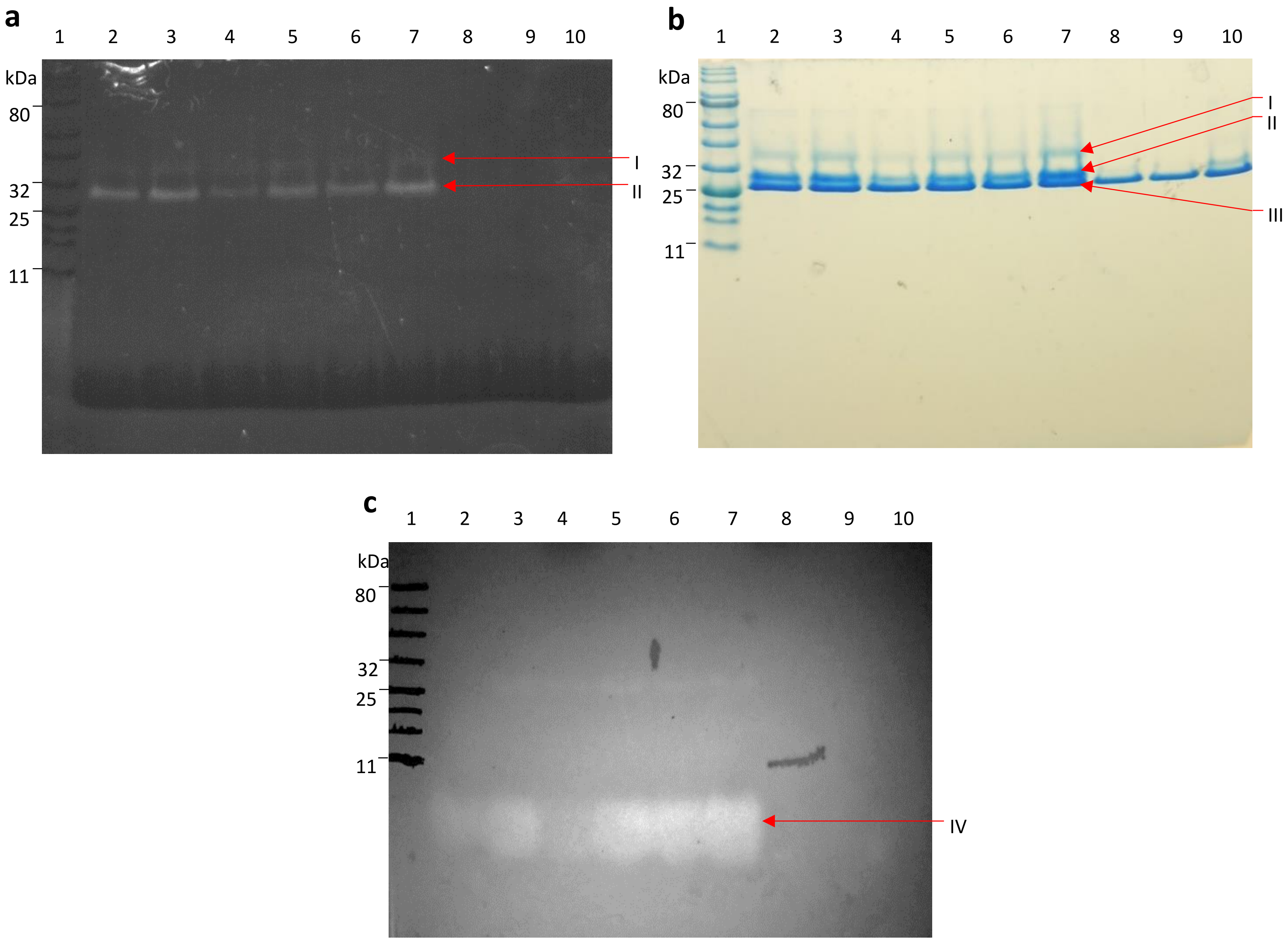

Supplementary Figure S2. SDS-PAGE analysis of Ni-NTA purified GFP-MunX from *E. coli* pRSF-GFP-MunX fermentations at 18°C, 26°C and 37 °C in triplicate. Lane: 1 – Ladder, 2 to 4 – biological triplicate fermentations at 18 °C, 5 to 7 – 26 °C, 8 to 10 – 37 °C. (**a**) Fluorometrically photographed gel where GFP fluorescence is shown as white bands. (**b**) Coomassie stained gel (**a**). (**c**) Overlay of duplicate gel (**a**) with agar seeded with *L. monocytogenes.* Bands: I – putative WELQut and GFP complex, II – uncleaved MunX, III – WELQut, IV – clear zone voids of *L. monocytogenes*.

6. Bonferroni posttests results for incubation temperature (For Fig. 2a)

Supplementary Table S3 Bonferroni posttests

**18 °C vs 26 °C**				
**Time**	**18 °C**	**26 °C**	**Difference**	**95% CI of diff.**
0	313,9	313,9	0,0000	-3497 to 3497
24	40310	39080	-1226	-4722 to 2271
48	40480	39090	-1389	-4885 to 2108
**Time**	**Difference**	**t**	**P value**	**Summary**
0	0,0000	0,0000	P > 0.05	ns
24	-1226	1,002	P > 0.05	ns
48	-1389	1,136	P > 0.05	ns
**18 °C vs 37 °C**				
**Time**	**18 °C**	**37 °C**	**Difference**	**95% CI of diff.**
**0**	**313,9**	**313,9**	**0,0000**	**-3497 to 3497**
24	40310	6388	-33920	-37410 to -30420
48	40480	5162	-35310	-38810 to -31820
**Time**	**Difference**	**t**	**P value**	**Summary**
0	0,0000	0,0000	P > 0.05	ns
24	-33920	27,73	P<0.001	***
48	-35310	28,88	P<0.001	***
**26 °C vs 37 °C**				
**Time**	**26 °C**	**37 °C**	**Difference**	**95% CI of diff.**
0	313,9	313,9	0,0000	-3497 to 3497
24	39080	6388	-32690	-36190 to -29200
48	39090	5162	-33930	-37420 to -30430
**Time**	**Difference**	**t**	**P value**	**Summary**
0	0,0000	0,0000	P > 0.05	ns
24	-32690	26,73	P<0.001	***
48	-33930	27,74	P<0.001	***

7. Tukeys multiple comparison test results for IPTG induction (For Fig. 3b)

Supplementary Table S4 Tukey's Multiple Comparison Test

Treatment	Mean Diff.	q	Significant? P < 0.05?	Summary	95% CI of diff
0.01 mM vs 0.05 mM	-709,6	16,84	Yes	***	-904.2 to -514.9
0.01 mM vs 0.1 mM	-971,9	23,06	Yes	***	-1166 to -777.3
0.01 mM vs 0.2 mM	-1074	25,48	Yes	***	-1268 to -879.2
0.01 mM vs 0.4 mM	-731,2	17,35	Yes	***	-925.8 to -536.6
0.01 mM vs 0.6 mM	-587,4	13,94	Yes	***	-782.1 to -392.8
0.01 mM vs 0.8 mM	-548,2	13,01	Yes	***	-742.8 to -353.6
0.01 mM vs 1.0 mM	-527,6	12,52	Yes	***	-722.2 to -332.9
0.01 mM vs 2.0 mM	-474,7	11,26	Yes	***	-669.3 to -280.1
0.01 mM vs Uninduced	341,1	8,093	Yes	***	146.5 to 535.7
0.05 mM vs 0.1 mM	-262,3	6,224	Yes	**	-456.9 to -67.73
0.05 mM vs 0.2 mM	-364,2	8,642	Yes	***	-558.8 to -169.6
0.05 mM vs 0.4 mM	-21,67	0,5141	No	ns	-216.3 to 172.9
0.05 mM vs 0.6 mM	122,1	2,897	No	ns	-72.50 to 316.7
0.05 mM vs 0.8 mM	161,3	3,828	No	ns	-33.27 to 355.9
0.05 mM vs 1.0 mM	182,0	4,318	No	ns	-12.61 to 376.6
0.05 mM vs 2.0 mM	234,9	5,573	Yes	**	40.28 to 429.5
0.05 mM vs Uninduced	1051	24,93	Yes	***	856.1 to 1245
0.1 mM vs 0.2 mM	-101,9	2,417	No	ns	-296.5 to 92.72
0.1 mM vs 0.4 mM	240,7	5,710	Yes	**	46.06 to 435.3
0.1 mM vs 0.6 mM	384,4	9,121	Yes	***	189.8 to 579.1
0.1 mM vs 0.8 mM	423,7	10,05	Yes	***	229.1 to 618.3
0.1 mM vs 1.0 mM	444,3	10,54	Yes	***	249.7 to 638.9
0.1 mM vs 2.0 mM	497,2	11,80	Yes	***	302.6 to 691.8
0.1 mM vs Uninduced	1313	31,15	Yes	***	1118 to 1508
0.2 mM vs 0.4 mM	342,6	8,128	Yes	***	147.9 to 537.2
0.2 mM vs 0.6 mM	486,3	11,54	Yes	***	291.7 to 680.9
0.2 mM vs 0.8 mM	525,6	12,47	Yes	***	330.9 to 720.2
0.2 mM vs 1.0 mM	546,2	12,96	Yes	***	351.6 to 740.8
0.2 mM vs 2.0 mM	599,1	14,21	Yes	***	404.5 to 793.7
0.2 mM vs Uninduced	1415	33,57	Yes	***	1220 to 1609
0.4 mM vs 0.6 mM	143,8	3,411	No	ns	-50.83 to 338.4
0.4 mM vs 0.8 mM	183,0	4,342	No	ns	-11.61 to 377.6
0.4 mM vs 1.0 mM	203,7	4,832	Yes	*	9.059 to 398.3
0.4 mM vs 2.0 mM	256,6	6,087	Yes	**	61.95 to 451.2
0.4 mM vs Uninduced	1072	25,44	Yes	***	877.7 to 1267
0.6 mM vs 0.8 mM	39,22	0,9306	No	ns	-155.4 to 233.8
0.6 mM vs 1.0 mM	59,89	1,421	No	ns	-134.7 to 254.5
0.6 mM vs 2.0 mM	112,8	2,676	No	ns	-81.83 to 307.4
0.6 mM vs Uninduced	928,6	22,03	Yes	***	733.9 to 1123
0.8 mM vs 1.0 mM	20,67	0,4903	No	ns	-173.9 to 215.3
0.8 mM vs 2.0 mM	73,56	1,745	No	ns	-121.1 to 268.2
0.8 mM vs Uninduced	889,3	21,10	Yes	***	694.7 to 1084
1.0 mM vs 2.0 mM	52,89	1,255	No	ns	-141.7 to 247.5
1.0 mM vs Uninduced	868,7	20,61	Yes	***	674.1 to 1063
2.0 mM vs Uninduced	815,8	19,36	Yes	***	621.2 to 1010

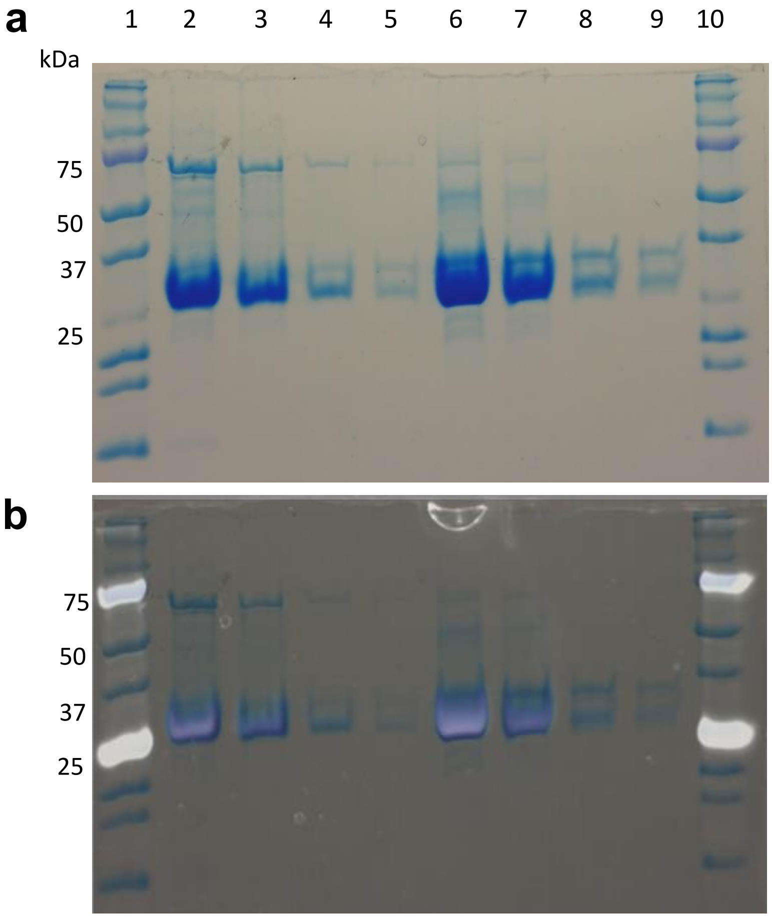

Supplementary Figure. S3 (**a**) Upscale expression and Ni-NTA purification of GFP-PlaX and GFP-MunX electrophoretically separated at various dilutions for purity estimation using Gel analyser. Lane: 1 - Ladder, 2 – GFP-PlaX undiluted, 3 - GFP-PlaX 2x diluted, 4 – GFP-PlaX 10x diluted, 5 – GFP-PlaX 20x diluted, 6 – GFP-MunX undiluted, 7 – GFP-MunX 2x diluted, 8 – GFP-MunX 10x diluted, 9 – GFP-MunX 20x diluted, 10 - Ladder. (**b**) Gel fluorometrically photographed and image super imposed on the stained gel. The fluorescence of GFP is observed as white bands in lane 2 – 9.

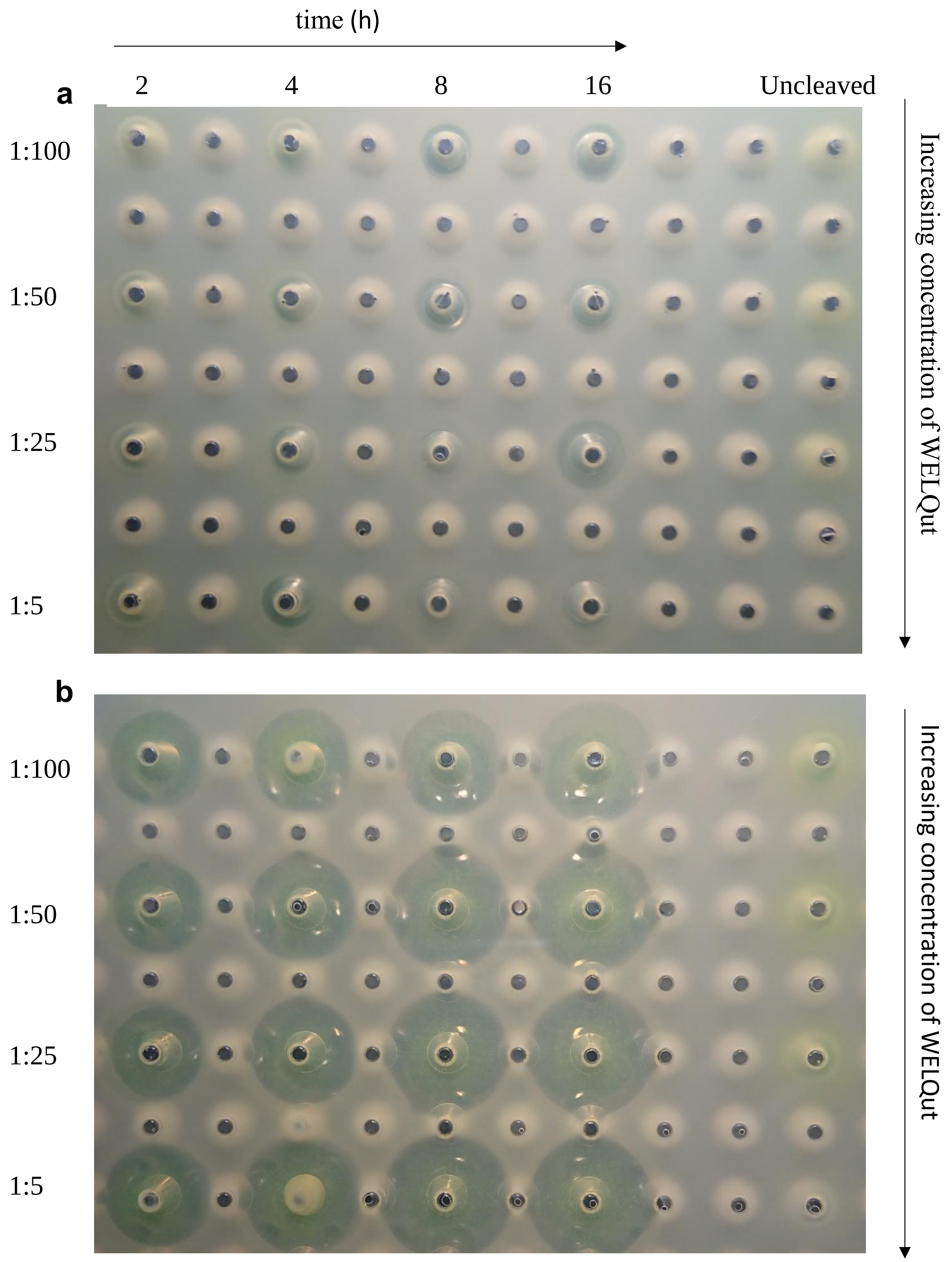

Supplementary Figure S4. WELQut cleavage optimisation of incubation time and WELQut **:** Sample ratio for maximal liberation of plantaricin 423 and mundticin ST4SA from Ni-NTA purified GFP-PlaX and GFP-MunX, respectively. Cleavage assessed using the spot plate technique against *L. monocytogenes*. (**a**) GFP-PlaX cleaved at 1:100, 1:50, 1:25, 1:5 WELQut **:** Sample ratios over time at 28 °C. (**b**) GFP-MunX cleaved at 1:100, 1:50, 1:25, 1:5 WELQut **:** sample ratios over time at 28 °C. Uncleaved GFP-PlaX (**a**) and GFP-MunX (**b**) did not show antilisterial activity. Zone diameters can be found in Table S5a

Supplementary Table S5a Zone diameter for Fig. S4

**GFP-PlaX (50 µL spot volume)**				
**Dilution**	**Time**			
	**2**	**4**	**8**	**16**
1:100	N/A	0,497	0,575	0,657
1:50	0,532	0,569	0,615	0,627
1:25	0,569	0,574	N/A	0,757
1:5	0,641	0,651	N/A	N/A
**GFP-MunX (50 µL spot volume)**				
**Dilution**	**Time**			
	**2**	**4**	**8**	**16**
1:100	1,035	1,096	1,119	1,399
1:50	1,044	1,228	1,384	1,458
1:25	1,048	1,188	1,383	1,435
1:5	1,188	1,315	1,47	1,416

Zone diameters measured in centimetres

Supplementary Table S5b Zone diameter for Fig. S4

**PlaX (100 µL spots volume)**	
1:100	0,669
1:50	0,84
1:25	0,942
1:10	1,04
**MunX (10 µL spots volume)**	
1:100	0,692
1:50	0,942
1:25	1,214
1:10	1,215

Zone diameters measured in centimetres

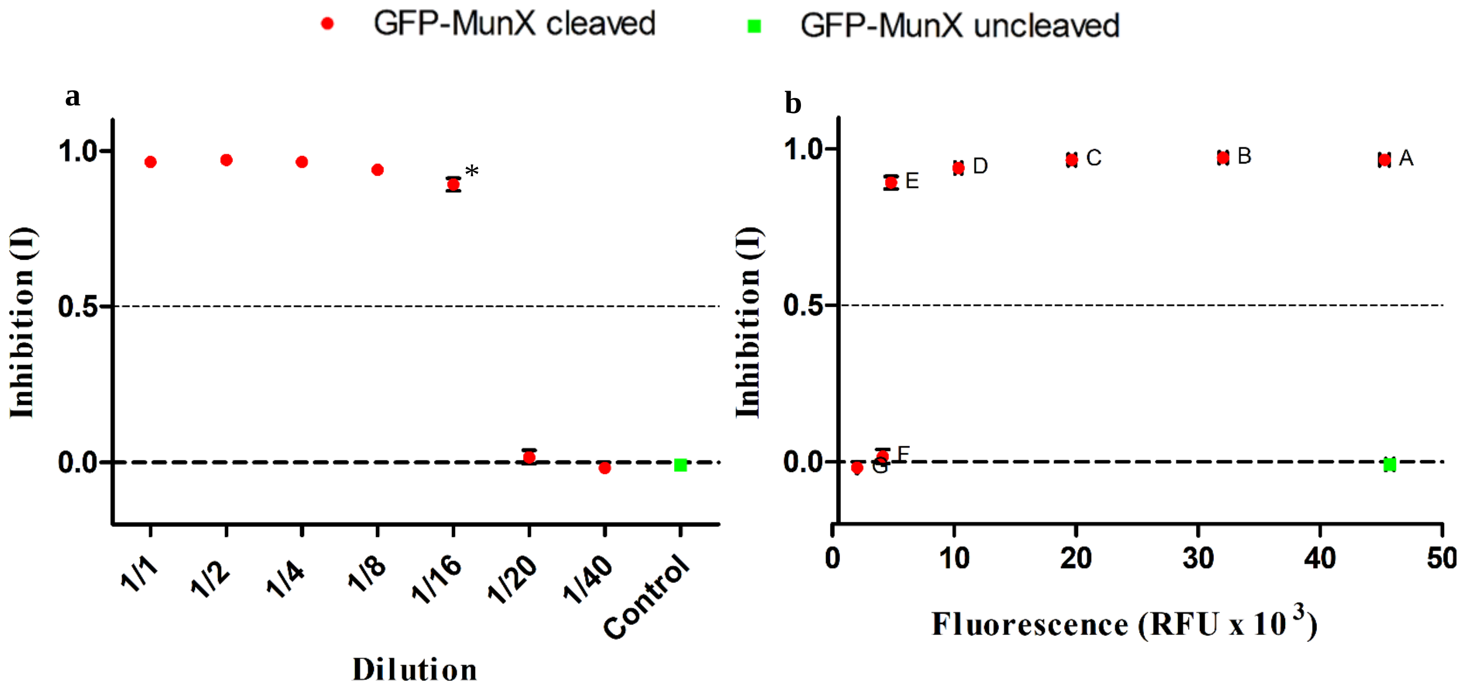

c

Dilution	Stock[#] (mg/mL)	Total protein (mg/mL)	Label[α]	RFUs x 10^3 (mean)	BU/mL[*]
1/1	10.95	17.96	A	45.27933	-
1/2	5.48	8.98	B	32.05267	-
1/4	2.74	4.49	C	19.63967	-
1/8	1.37	2.25	D	10.35400	-
1/16	0.68	1.12	E	4.824000	1600
1/20	0.55	0.90	F	4.146333	-
1/40	0.27	0.45	G	2.047333	-
1/1 (Control)	10.95	17.96	Control	45.71133	-

*Bacteriocin units/mL. #From SDS-PAGE analysis of purity. α For figure S5b

Supplementary Figure S5. a) Minimum inhibitory concentration analysis of mundticin ST4SA, liberated from GFP-MunX. Listerial inhibition was expressed as I = 1 – (A_m/A_0), where A_m is the sample absorbance and A_0 the control absorbance at 595nm. Points represent means (n=3) and SEM indicated by error bars. Asterisk (*) indicates the point used to calculate bacteriocin units (BU). **b)** Fluorescent intensities of serial dilutions for mundticin ST4SA, liberated from GFP-MunX, versus their ability to inhibit the growth of listeria. Listerial inhibition was expressed as in **(a)**. Each point represents the calculated RFU (n=3, x axis) and inhibition (n=3, y axis) means with vertical and horizontal SEM indicated by error bars. The concentrations of each sample in the serial dilution is represented in **(c)**. Uncleaved GFP-MunX served as the control.

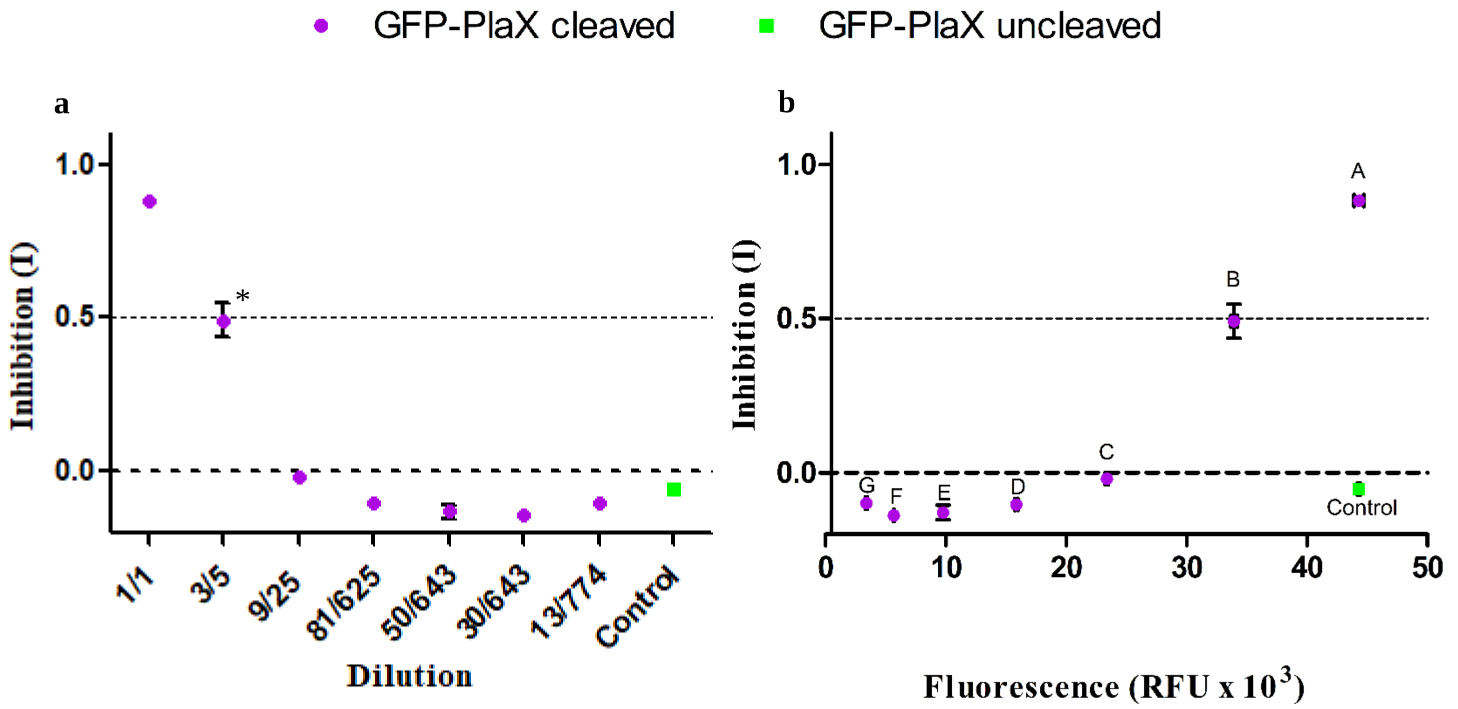

c

Dilution	Stock# (mg/mL)	Total protein (mg/mL)	Label$^{\alpha}$	RFUs x 10^3 (mean)	BU/mL*
1/1	9.33	12.96	A	44.31700	-
3/5	5.60	7.78	B	33.94300	83.33
9/25	3.36	4.67	C	23.36467	-
81/625	1.21	1.68	D	15.85633	-
50/643	0.73	1.01	E	9.77433	-
30/643	0.44	0.60	F	5.67900	-
13/774	0.16	0.22	G	3.40200	-
1/1 (Control)	9.33	12.96	Control	44.31700	-

*Bacteriocin units/mL. #From SDS-PAGE analysis of purity. α for figure S5b

Supplementary Figure S6. a) Minimum inhibitory concentration analysis of plantaricin 423, liberated from GFP-PlaX. Listerial inhibition was expressed as $I = 1 – (A_m/A_0)$, where A_m is the sample absorbance and A_0 the control absorbance at 595nm. Points represent means (n=3) and SEM indicated by error bars. Asterisk (*) indicates the point used to calculate bacteriocin units (BU). **b)** Fluorescent intensities of serial dilutions for plantaricin 423, liberated from GFP-PlaX, versus their ability to inhibit the growth of listeria. Listerial inhibition was expressed as in **(a)**. Each point represents the calculated RFU (n=3, x axis) and inhibition (n=3, y axis) means with vertical and horizontal SEM indicated by error bars. The concentrations of each sample in the serial dilution is represented in **(c)**. Uncleaved GFP-PlaX served as the control.

a

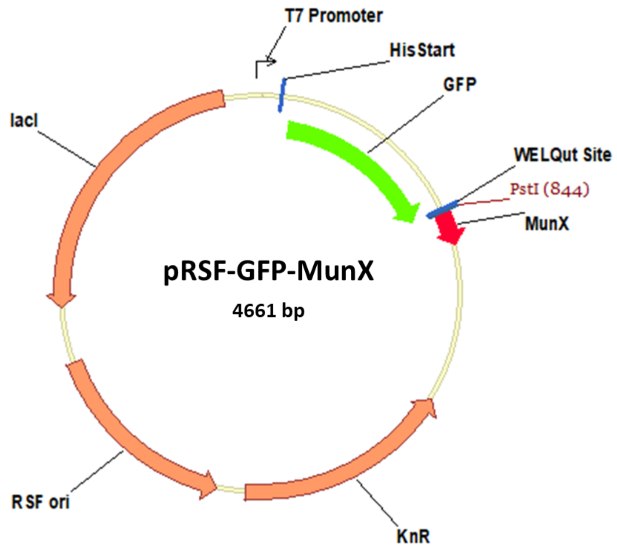


```
                                                                                   GFP-MunX
                                                                                   ------->
                                                                                  M  G  S  S   H  H  H   H  H  H
  1 GGGGAATTGT GAGCGGATAA CAATTCCCCT GTAGAAATAA TTTTGTTTAA CTTTAATAAG GAGATATACC ATGGGCAGCA GCCATCACCA TCATCACCAC
    CCCCTTAACA CTCGCCTATT GTTAAGGGGA CATCTTTATT AAAACAAATT GAAATTATTC CTCTATATGG TACCCGTCGT CGGTAGTGGT AGTAGTGGTG
     S  Q  D  P   M  S  K   G  E  E   L  F  T  G   V  V  P   I  L  V   E  L  D  G   D  V  N   G  Y  K   X  X  X  X ·
101 AGCCAGGATC CGATGAGTAA AGGAGAAGAA CTTTTCACTG GAGTTGTCCC AATTCTTGTT GAATTAGATG GTGATGTTAA TGGGTACAAA    //
    TCGGTCCTAG GCTACTCATT TCCTCTTCTT GAAAAGTGAC CTCAACAGGG TTAAGAACAA CTTAATCTAC CACTACAATT ACCCATGTTT   //
                                                   PstI    Mundticin ST4SA
                                                ~~~~~~~~ ↓  ------->
      H  G  M   D  E  L   Y  K  T  G   W  E  L   Q K  Y   Y  G  N  G   V  S  C   N  K  K   G  C  S  V   D  W  G ·
801 CACATGGCAT GGATGAACTA TACAAAACCG GTTGGGAACT GCAGAAATAC TACGGTAATG GAGTCTCATG TAATAAAAAA GGGTGCAGTG TTGATTGGGG
    GTGTACCGTA CCTACTTGAT ATGTTTTGGC CAACCCTTGA CGTCTTTATG ATGCCATTAC CTCAGAGTAC ATTATTTTTT CCCACGTCAC AACTAACCCC
     K  A  I   G  I  I  G   N  N  S   A  A  N   L  A  T  G   G  A  A   G  W  K   S  *
901 AAAAGCTATT GGCATTATTG GAAATAATTC TGCTGCGAAT TTAGCTACTG GTGGAGCAGC TGGTTGGAAA AGTTAA
    TTTTCGATAA CCGTAATAAC CTTTATTAAG ACGACGCTTA AATCGATGAC CACCTCGTCG ACCAACCTTT TCAATT
```

Supplementary Figure S7a. Plasmid map of pRSF-GFP-MunX for the T7 controlled heterologous expression of GFP-MunX. Liberation of mundticin ST4SA (red) using the WELQut protease with cleavage sequence as indicated (grey arrow) in the amino acid sequence for GFP-MunX.

b

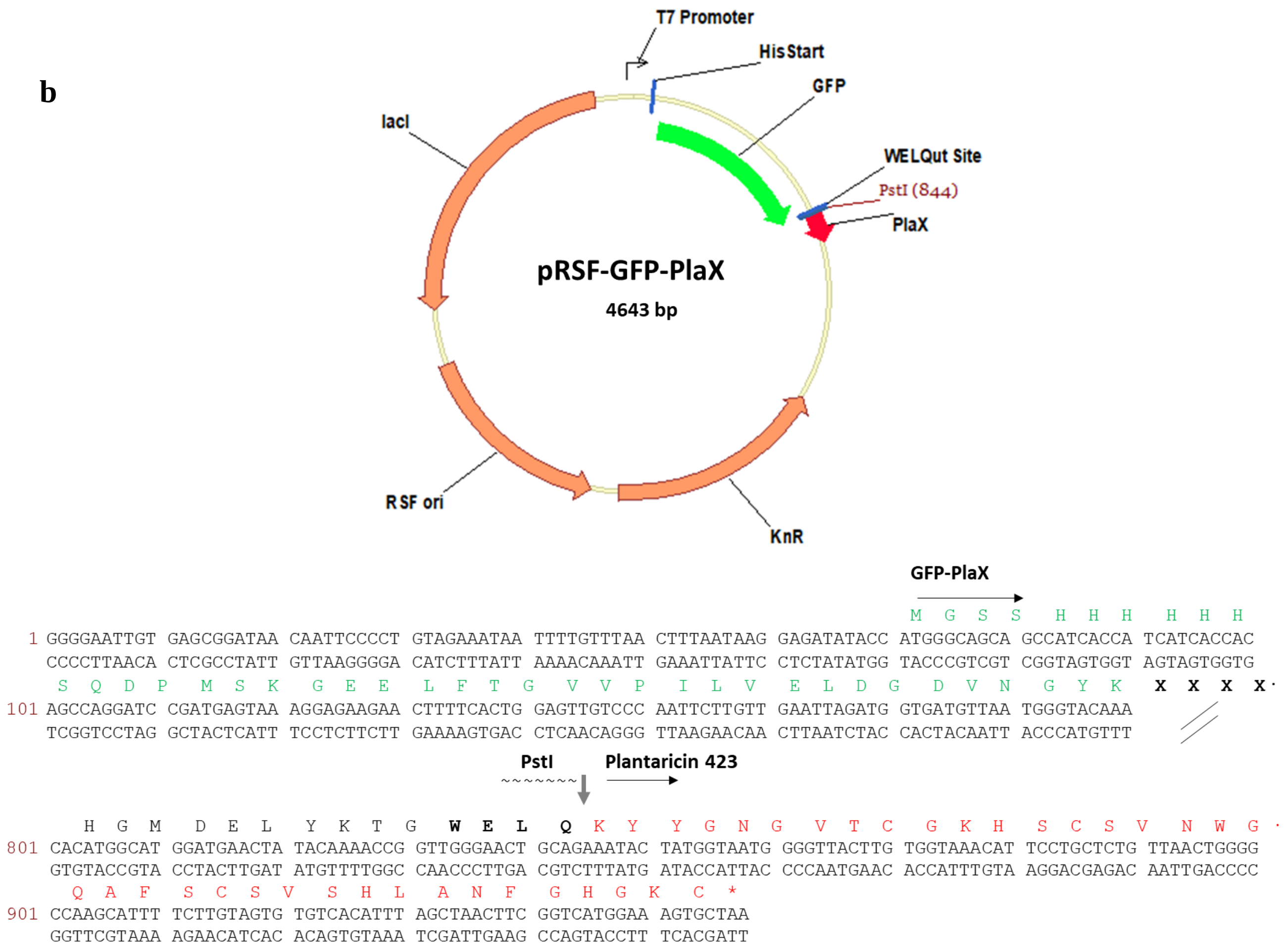

Supplementary Figure S7b. Plasmid map of pRSF-GFP-PlaX for the T7 controlled heterologous expression of GFP-PlaX. Liberation of plantaricin 423 (red) using the WELQut protease with cleavage sequence as indicated (grey arrow) in the amino acid sequence for GFP-PlaX.

Supplementary Table S6 DNA oligonucleotide sequences

GFP_Bam_Fwd	5’-GGATCCGATGAGTAAAGGAGAAGAACTTTTCACTGGAGTTGTCCCAATTC-3’
GFP_WELQ_Rev	5’-CTGCAGTTCCCAACCGGTTTTGTATAGTTCATCCATGCCATGTGTAATCC-3’
GFP-PlaX_Pst_Fwd	5’- TAAGGGATCCGTGGGAACTGCAGAAATACTATG-3’
GFP-PlaX_Hind_Rev	5’-TATTAAGCTTAGCACTTTCCATGACCGAAGTTAGCTAAATG-3’
GFP-MunX_Pst_Fwd	5’-ATCGCTGCAGAAATACTACGGTAATGGAGTCTCATGTAATAAAAAAG-3’
GFP-MunX_Hind_Rev	5’- ACGCAAGCTTAACTTTTCCAACCAGCTGC-3’
pJET1.2_Fwd	5’- CGACTCACTATAGGGAGAGCGGC-3’
pJET1.2_Rev	5’- AAGAACATCGATTTTCCATGGCAG-3’
MCS1_Rev	5’-GATTATGCGGCCGTGTACAA-3’

Supplementary Table S7 Buffers used in IMAC purification and WELQut cleavage

SB	50 mM Tris, 500 mM NaCl, 10% glycerol (v/v) pH8.0
SB500	50 mM Tris, 500 mM NaCl, 500 mm imidazole, 10% glycerol (v/v) pH8.0
WELQut cut buffer	100 mM Tris, pH 8.0
10X TB buffer	0.17 M KH_2PO_4, 0.72 M K_2HPO_4

Supplementary Table S8 Strains and vectors used in this study

Strain	Characteristic	Ref
Escherichia coli BL21 (DE3)	Expression host	*
Lactobacillus plantarum 423	Plantaricin 423 producer	1
Enterococcus mundtii ST4SA	Mundticin ST4SA producer	2
Listeria monocytogenes EDG-e	Sensitive strain	*
**Plasmid**	**Characteristic**	**Ref**
pRSF Duet-1	Vector with the IPTG inducible P_{T7}, Km^R and cloning site for N-terminal His tag fusion.	Ꙩ
pTRKH3-ermGFP	Plasmid containing GFP, Ery^R	Θ; 3
pJET-GFP	GFP-cloning vector	ϕ
pRSF-GFP-PlaX	Heterologous expression of GFP-PlaX	This study
pRSF-GFP-MunX	Heterologous expression of GFP-MunX	This study

* Lab culture collection. Θ Gift from Michela Lizier, Addgene plasmid #27169; http://n2t.net/addgene:27169. Ꙩ Novagen. ϕ Thermo Fisher Scientific.

1 (van Reenen et al., 1998); 2 (Granger et al., 2008); 3 (Lizier et al., 2010)

8. References

Granger, M., van Reenen, C. A., and Dicks, L. M. T. (2008). Effect of gastro-intestinal conditions on the growth of *Enterococcus mundtii* ST4SA, and production of bacteriocin ST4SA recorded by real-time PCR. *Int. J. Food Microbiol.* 123, 277–280. doi:10.1016/j.ijfoodmicro.2007.12.009.

Lizier, M., Sarra, P. G., Cauda, R., and Lucchini, F. (2010). Comparison of expression vectors in *Lactobacillus reuteri* strains. *FEMS Microbiol. Lett.* 308, 8–15. doi:10.1111/j.1574-6968.2010.01978.x.

van Reenen, C. A., Dicks, L. M. T. and Chikindas, M. L. (1998). Isolation, purification and partial characterization of plantaricin 423, a bacteriocin produced by *Lactobacillus plantarum*. *J. Appl. Microbiol.* 84, 1131–1137. doi:10.1046/j.1365-2672.1998.00451.x.
